# A bacterial pioneer produces cellulase complexes that persist through community succession

**DOI:** 10.1038/s41564-017-0052-z

**Published:** 2017-11-06

**Authors:** Sebastian Kolinko, Yu-Wei Wu, Firehiwot Tachea, Evelyn Denzel, Jennifer Hiras, Raphael Gabriel, Nora Bäcker, Leanne Jade G. Chan, Stephanie A. Eichorst, Dario Frey, Qiushi Chen, Parastoo Azadi, Paul D. Adams, Todd R. Pray, Deepti Tanjore, Christopher J. Petzold, John M. Gladden, Blake A. Simmons, Steven W. Singer

**Affiliations:** 1 Joint BioEnergy Institute, Emeryville, CA USA; 2 Biological Systems and Engineering Division, Lawrence Berkeley National Laboratory, Berkeley, CA USA; 3 Graduate Institute of Biomedical Informatics, College of Medical Science and Technology, Taipei Medical University, Taipei, Taiwan; 4 Advanced Biofuels Process Development Unit, Lawrence Berkeley National Laboratory, Emeryville, CA USA; 5 Physical Biosciences Division, Lawrence Berkeley National Laboratory, Berkeley, CA USA; 6 Faculty of Biotechnology, University of Applied Sciences, Mannheim, Germany; 7 Institut für Genetik, Technische Universität Braunschweig, Braunschweig, Germany; 8 Complex Carbohydrate Research Center, University of Georgia, Athens, GA USA; 9 Molecular Biophysics and Integrated Bioimaging Division, Lawrence Berkeley National Laboratory, Berkeley, CA USA; 10 Biological and Materials Science Center, Sandia National Laboratories, Livermore, CA USA; 11 Present Address: Department of Chemistry, University of Basel, Basel, Switzerland; 12 Present Address: Corning Incorporated, Corning, NY USA; 13 Present Address: Division of Microbial Ecology, Department of Microbiology and Ecosystem Science, Research network “Chemistry meets Microbiology”, University of Vienna, Vienna, Austria

**Keywords:** Metagenomics, Environmental microbiology, Bacterial genomics

## Abstract

Cultivation of microbial consortia provides low-complexity communities that can serve as tractable models to understand community dynamics. Time-resolved metagenomics demonstrated that an aerobic cellulolytic consortium cultivated from compost exhibited community dynamics consistent with the definition of an endogenous heterotrophic succession. The genome of the proposed pioneer population, ‘*Candidatus* Reconcilibacillus cellulovorans’, possessed a gene cluster containing multidomain glycoside hydrolases (GHs). Purification of the soluble cellulase activity from a 300litre cultivation of this consortium revealed that ~70% of the activity arose from the ‘*Ca*. Reconcilibacillus cellulovorans’ multidomain GHs assembled into cellulase complexes through glycosylation. These remarkably stable complexes have supramolecular structures for enzymatic cellulose hydrolysis that are distinct from cellulosomes. The persistence of these complexes during cultivation indicates that they may be active through multiple cultivations of this consortium and act as public goods that sustain the community. The provision of extracellular GHs as public goods may influence microbial community dynamics in native biomass-deconstructing communities relevant to agriculture, human health and biotechnology.

## Abstract (web summary)

Cultivation of a cellulolytic consortium reveals successional community dynamics and the presence of multidomain glycoside hydrolases assembled into stable complexes distinct from cellulosomes, which are produced by a potential pioneer population.

Plant polysaccharide hydrolysis is a critical process in the human microbiome^1^, soil microbiomes^2^ and microbiomes related to bioenergy production^3–5^. Identifying glycoside hydrolases (GHs) responsible for polysaccharide hydrolysis in these ecosystems has important implications for improving human health, managing agriculture and implementing biotechnological advances. Characterizing GHs from uncultivated organisms may also expand the diversity of protein structures that hydrolyse polysaccharides^6^. However, these communities harbour substantial diversity, complicating the assignment of specific enzymatic roles to individual community members.

Model microbial consortia with simplified community compositions relative to native consortia have been identified as important systems to develop a mechanistic understanding of community function^7^. Methods to cultivate these model consortia include combining isolates from native consortia and adapting native communities through selective pressure^8^. These model consortia have enabled the assignment of function to specific microbial community members, clarified successional structures in communities^9^ and identified previously unknown protein functions^10^. Cultivation of model consortia that hydrolyse cellulose, the most abundant plant polysaccharide^11^, has produced low-complexity communities where cellulose hydrolysis can be assigned to specific populations and linked to community structure and dynamics^12,13^.

Here, we report that a model cellulolytic consortium derived from compost was reproducibly cultivated aerobically at 15 l and 300 l. The proposed pioneer population^7^ in this community, present at ~1% abundance at the time of culture harvest, produced multidomain cellulases that persisted through microbial succession and were the most active cellulases in the culture. These cellulases were organized in protein complexes that are distinct from cellulosomes^8^ isolated from anaerobic bacteria. The persistence of these unusually stable complexes indicates that they may act as public goods^14^ that sustain the community.

## Results

**Fig. 1:**
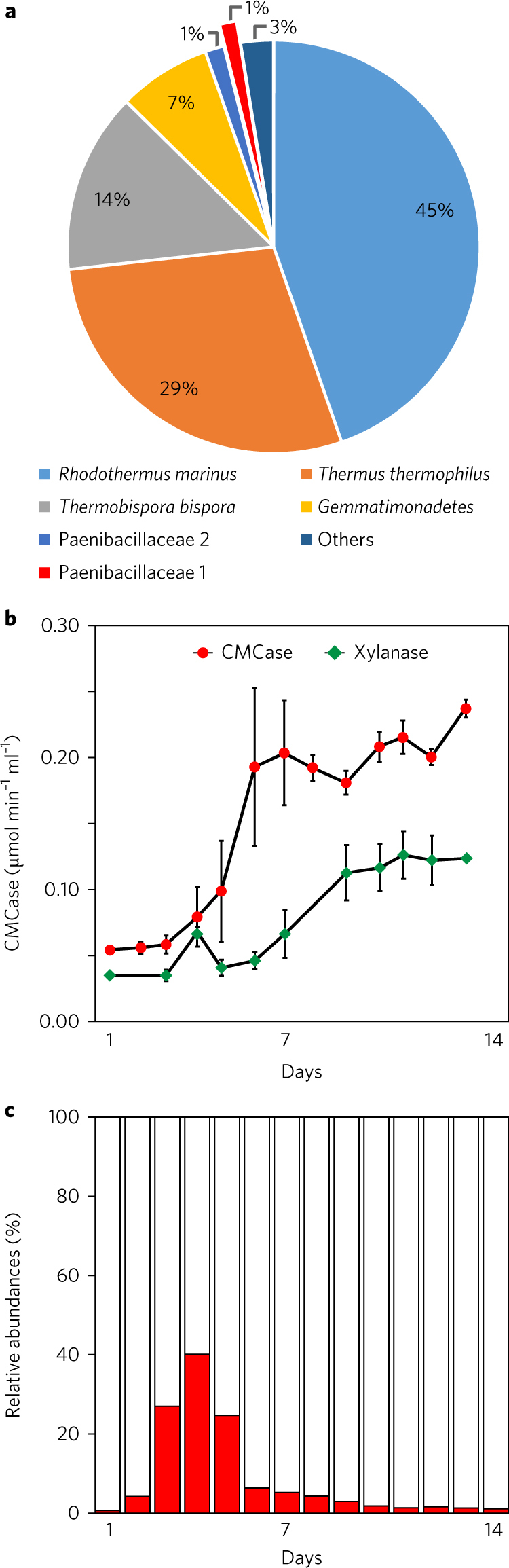
Cultivation of cellulolytic consortium at 15 l scale. **a**, Relative abundance of dominant populations (≥1%) at the end of the cultivation. Detailed genome information and average coverage are provided in Supplementary Tables 1 and 2. Single DNA samples were isolated from the 15 l culture on each indicated day for metagenomic sequencing. **b**, CMCase (red) and xylanase (green) activity measurements obtained by daily sampling of the 15 l cultivation. Enzymatic assays are reported as the mean of technical replicates (*n* = 3) and error bars represent standard error of the mean. **c**, Daily relative abundances (calculated using the average number of reads of binned scaffolds from time-series metagenomic data for the 15 l cultivation) of the Paenibacillaceae 1 population (red) during a 14-day cultivation of the consortium grown with microcrystalline cellulose.

A thermophilic cellulolytic bacterial consortium cultivated aerobically at 60 °C from compost obtained from Vacaville, CA, USA produced extracellular cellulases that released glucose from pretreated plant biomass at temperature up to 80 °C^15,16^. This consortium was maintained for >3 years by passaging every 2 weeks in 50 ml shake flasks. Cultivation of the consortium was scaled to 15 l, and the culture from this 15 l bioreactor was used to inoculate a 300 l bioreactor to provide enzymes for bioprocess studies of biomass deconstruction^17^ and to purify individual soluble cellulases. The dynamics of the consortium were analysed by time-resolved metagenomics and population genomes recovered by automated binning. At the end of the cultivation, the most abundant populations in the 15 l cultivation were closely related to *Rhodothermus marinus* (45%), *Thermus thermophilus* (29%) and *Thermobispora bispora* (14%) (Fig. 1a and Supplementary Tables 1 and 2). The 300 l cultivation had a similar community composition, with a lower proportion of *T. thermophilus* (Supplementary Fig. 1a and Supplementary Tables 3 and 4). CMCase activity, a proxy for cellulase activity, increased between days 4 and 5 (from ~0.03–0.05 µmol ml^−1^ min^−1^ to ~0.2 µmol ml^−1^ min^−1^) and levelled off in both cultivations, while the xylanase activity showed divergent behaviour (Fig. 1b and Supplementary Fig. 1b). Surprisingly, in the 15 l cultivation, CMCase activity correlated with the population dynamics of a single population, affiliated with the Paenibacillaceae (Paenibacillaceae 1; Bin 008). This population rapidly increased in relative abundance from day 2 (4%) to day 4 (40%), but diminished to below 5% by day 8 (Fig. 1c). Similar dynamics were observed in a more restricted sampling of the 300 l cultivation (Supplementary Fig. 1c). The dynamic behaviour of the Paenibacillaceae 1 population is consistent with a pioneer population that commences a community succession^7^. This succession resembles the model for endogenous heterotrophic succession described by Fierer et al.^18^, in which the Paenibacillaceae 1 population responded rapidly but was outcompeted by other populations, particularly *R. marinus*, which becomes the most abundant population in both bioreactor experiments as well as previous cultivations of this consortium in shake flasks^12,16^. These compositional profiles demonstrated the cellulolytic consortium displayed reproducible behaviour at volumes from 50 ml to 300 l.

**Fig. 2:**
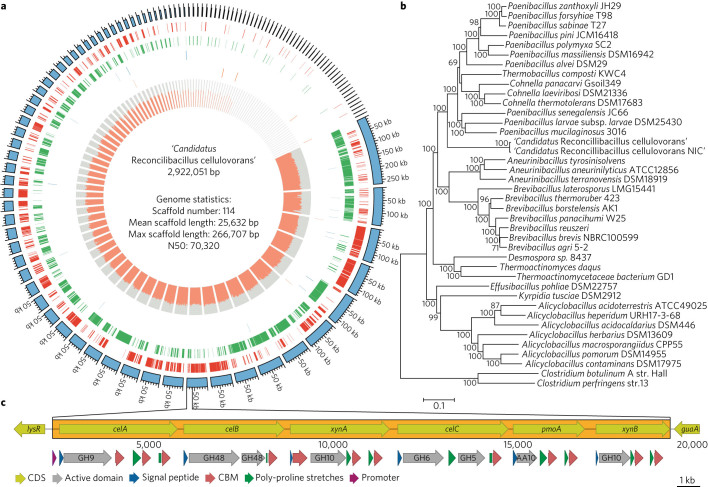
Genome analysis. **a**, Population genome of ‘*Ca.* R. cellulovorans’ recovered from metagenomics data from the 15 l cultivation. The genome was dispersed on 114 scaffolds (blue), with 2,814 predicted CDS (coding DNA sequences) in forward (red) and reverse (green) and average (orange) coverage. N50 is the shortest sequence length that includes 50% of the assembled genome, summing from the largest contig. **b**, Maximum-likelihood phylogenetic tree based on 86 concatenated amino acid sequences that are conserved in the Paenibacillaceae (Supplementary Table 6). **c**, Molecular organization of multidomain GH genes from the population genome of ‘*Ca*. R. cellulovorans’ arranged in a 17-kb gene cluster.

Phylogenetic trees constructed from conserved proteins and rRNA genes recovered from the population genome of the Paenibacillaceae 1 bin demonstrated that this population is distinct from isolates in the Paenibacillaceae and represents a distinct genus (Fig. 2a,b, Supplementary Fig. 2 and Supplementary Tables 5 and 6). The species represented by this population was named ‘*Candidatus* Reconcilibacillus cellulovorans’ (from reconciliare in Latin, present active infinitive, to recover) because of evidence that this population is closely related to an incompletely characterized cellulolytic isolate, ‘Caldibacillus cellulovorans’, based on the similarity of its DNA polymerase (Supplementary Fig. 3)^19,20^. The genome of ‘*Ca*. R. cellulovorans’ is distantly related to sequenced Paenibacillaceae isolates (<70% amino acid identity). However, a nearly identical population genome was recovered from the metagenome of a similar adaptation of compost microbiota from Milipitas, CA, USA to grow on crystalline cellulose at 60 °C (Supplementary Table 7)^13^. This ‘*Ca*. R. cellulovorans NIC (Newby Island Compost)’ population was at <1% relative abundance after a two-week cultivation. Time-series analysis of this consortium using 16S rRNA marker genes provided evidence for a succession transitioning from Paenibacillaceae populations, including operational taxonomic units (OTUs) > 99% identical to the 16S rRNA gene sequence of ‘*Ca*. R. cellulovorans’, to a thermophilic population in the Chitinophagaceae. The importance of succession in this consortium was underscored by the inability of an isolated representative of the Chitinophagaceae population, strain NYFB, to grow with insoluble cellulose as the sole carbon source.

Analysis of the genome of ‘*Ca*. R. cellulovorans’ focused on its glycoside hydrolases (GHs) to assess its role in cellulose depolymerization. The initial population genome contained six partial genes on small discontinuous contigs encoding for multidomain GHs containing catalytic subunits that were linked to family 3 CBMs (CBM3b and CBM3c). PCR-based reconstruction demonstrated that these genes formed a 17-kb gene cluster containing multidomain GHs and a lytic polysaccharide monooxygenase (LPMO) (Fig. 2c, Supplementary Fig. 4 and Supplementary Table 8). The multidomain structure of the encoded proteins in this gene cluster resembled multidomain cellulases identified in the genomes of *Caldicellulosiruptor* isolates, a genus of extremely thermophilic, anaerobic bacteria that also contain multidomain GHs (Supplementary Fig. 5)^21^. However, the ‘*Ca*. R. cellulovorans’ GHs were capped at the C terminus by a CBM3 domain, while many of the *Caldicellulosiruptor* GHs are multidomain proteins that contain multiple catalytic domains linked by CBM3s^22,23^. The ‘*Ca*. R. cellulovorans’ CelA has a GH9 catalytic domain directly linked to a CBM3c and two CBM3b linked to the GH9-CBM3c sequence through poly-proline linkers. The N-terminal portion of *Caldicellulosiruptor bescii* CelA has an identical domain structure to ‘*Ca*. R. cellulovorans’ CelA, with 58% sequence identity, but has an additional GH48 domain appended to the C terminus. Despite the similarity in domain structure and sequence between ‘*Ca*. R. cellulovorans’ and *C. bescii* CelA, a phylogenetic tree of the catalytic domains demonstrated that the GH9 catalytic domain of these proteins clustered with members of their own phylogenetic group, which was not consistent with horizontal gene transfer (Supplementary Fig. 6a). Multidomain GHs with GH9 catalytic domains have been identified in members of the Paenibacillaceae, exemplified by the GH9 from *Paenibacillus barcinonensis*, which has a GH9 domain directly linked to a CBM3c, identical to ‘*Ca*. R. cellulovorans’ CelA, but has a fibronectin-like III domain and a CBM3b domain at the C terminus, rather than two CBM3b domains as in CelA^24^. In ‘*Ca*. R. cellulovorans’, the downstream gene CelB contains a GH48 domain that is 68 amino acid residues longer than the *Caldicellulosiruptor* GH48 domains (Supplementary Fig. 6b). XynA contains two CBM3b modules and an N-terminal GH10 catalytic domain that is 99% identical to a GH10 domain of a xylanase from ‘C. cellulovorans’ (Supplementary Fig. 6c)^25^. CelC contains an N-terminal GH6 domain and a C-terminal GH5 domain, with both domains clustering with members of the Paenibacillaceae (Supplementary Fig. 6d,e). The N-terminal AA10 domain of PmoA clustered with the N-terminal domain of ManA of ‘C. cellulovorans’ (>99% amino acid identity) (Supplementary Fig. 6f).

**Fig. 3:**
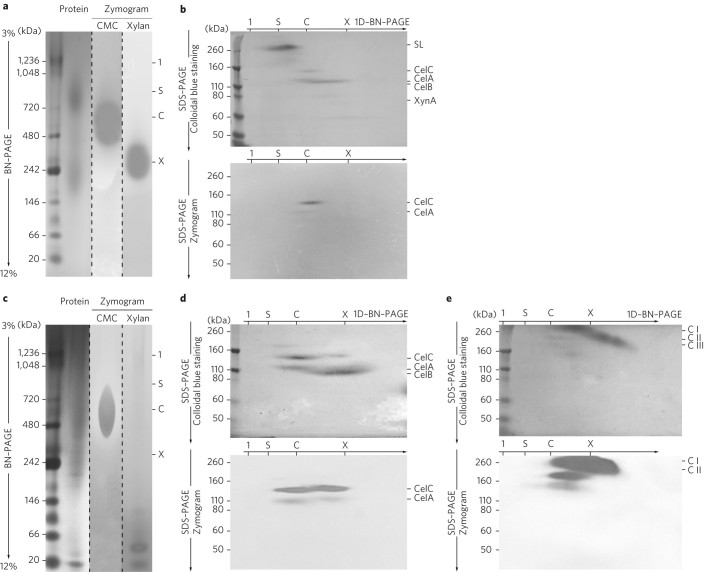
Analysis of GH complexes. **a**, Separation of cellulase and xylanase complexes eluted from an anion-exchange chromatography column at 260 mM NaCl and visualized by 2D BN–PAGE^58^. Complexes containing S-Layer proteins (S), CMCase (C) and xylanase (X) activity were separated in the first dimension according to their indicated masses by BN–PAGE. Protein staining was accompanied by zymography with gels embedded with CMC and xylan. **b**, Subunits of the native complexes were separated in a second dimension by SDS–PAGE (8%) and identified as CelABC and XynA by proteomics. The XynA molecular weight (~80 kDa) is indicative of a truncation of the full-length protein (100 kDa). Zymography with CMC revealed the activity of bands corresponding to CelC and CelA. **c**, GH complexes enriched by affinity digestion were separated by BN–PAGE and protein stains were accompanied by zymography with gels embedded with CMC and xylan. **d**, Native complexes were separated by SDS–PAGE into subunits CelABC and visualized by protein and CMCase activity staining. **e**, SDS–PAGE was also performed without initial heat denaturation, and three abundant individual complexes with different compositions of CelABC were identified by proteomics. Detailed proteomics data are provided in Supplementary Figs. 8 and 9. Images were cropped for clarity. Each gel is representative of five gels performed on multiple protein preparations. Gels stained with Coomassie and analysed by zymography were run in parallel in the same electrophoretic cell to ensure comparability. The gels in this figure are from one individual protein preparation for each of the two purification techniques described in the text. The gel images were cropped for clarity and the original gel images are provided in Supplementary Fig. 14.

**Fig. 4:**
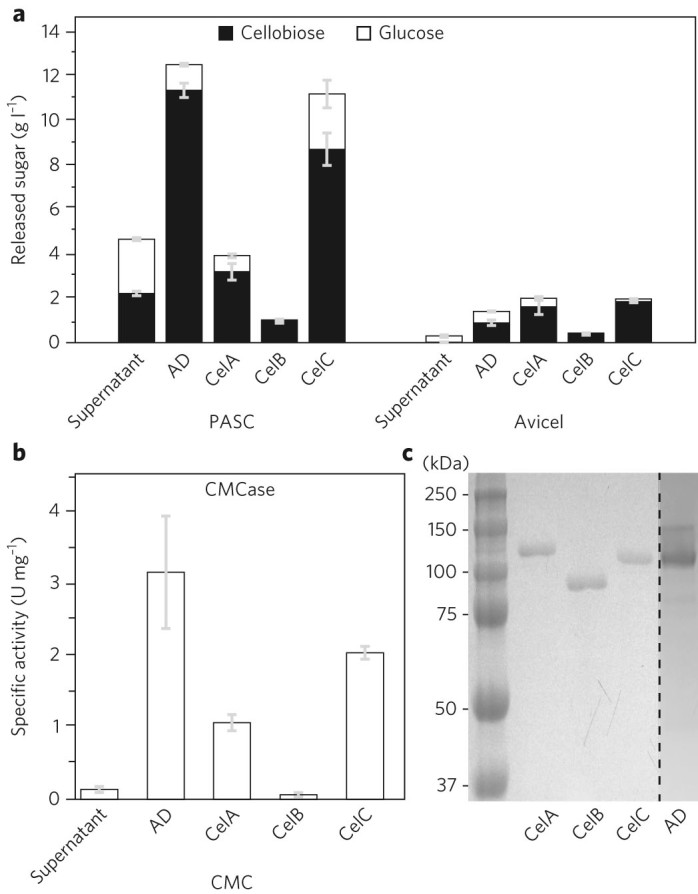
Cellulolytic activities of CelABC. **a**, Saccharification of phosphoric acid swollen cellulose (PASC) and Avicel for the culture supernatant, affinity digested preparation (AD) and recombinant CelABC individually expressed in *E. coli* (see Methods for details). Enzymatic assays are reported as the mean of technical replicates (*n* = 3) and error bars represent standard error of the mean. **b**, CMCase-specific activities of the supernatant, AD fraction and in *E. coli*-expressed CelABC. Enzymatic assays are reported as the mean of technical replicates (*n* = 3) and error bars represent standard error of the mean. **c**, SDS–PAGE (8–16% gradient; stained with Coomassie Brilliant Blue) of the AD fraction and *E. coli*-expressed CelABC. The gel depicted is representative of three gels that displayed very similar results. The gel images were cropped for clarity; the original gel images are provided in Supplementary Fig. 15.

Purification of the active GH components from the 300 l cultivation was performed to determine whether the ‘*Ca*. R. cellulovorans’ proteins were present in the supernatant. The majority (66.3% CMCase and 54.1% xylanase) of the GH activity eluted from an anion-exchange column at 260 mM NaCl (Supplementary Fig. 7a). Visualization and activity staining demonstrated that the GH activities arose from two broad protein bands with molecular weights centred at ~600 kDa (CMCase) and ~300 kDa (xylanase) (Fig. 3a). Separation of these broad bands by SDS–PAGE and subsequent proteomics analysis (Fig. 3b, Supplementary Figs. 8 and 9a–d and Supplementary Tables 9 and 10a–d) indicated that these bands represented protein complexes containing CelABC and XynA (bound to CelB) from ‘*Ca*. R. cellulovorans’. In an alternative method, the supernatant was treated by affinity digestion to remove proteins that lacked the capacity to bind and hydrolyse insoluble cellulose^26^. The affinity digestion procedure provided a protein preparation in which the CelABC complexes were enriched but the CelB–XynA complex was not recovered (Fig. 3c). Anion exchange chromatography of the affinity-digested preparation confirmed the retention of CMCase activity and loss of most of the xylanase activity (Supplementary Fig. 7b). A second dimension SDS–PAGE of the heat-denatured samples demonstrated that the CelABC formed three distinct complexes: a high-molecular-weight complex primarily containing CelC and CelA, a lower-molecular-weight complex containing CelABC, and a homocomplex containing only CelB (Fig. 3d). These three complexes were also visualized by denaturing the samples at room temperature and separating them by SDS–PAGE, which demonstrated that the complexes remained intact in the presence of 2% SDS (Fig. 3e). Zymography established that the CelC- and CelA-containing complexes accounted for the CMCase activity of the preparation, while no CelB activity was evident in the gel. The affinity-digested preparation had higher hydrolytic activity on carboxymethylcellulose (CMC) (~25×), phosphoric acid-swollen cellulose (PASC) (~3×) and Avicel (~3×) compared to the original supernatant (Fig. 4a,b).

Characterization of recombinant CelABC was performed to determine their cellulase activities. *E. coli*-expressed CelB and CelC were ~10–50 kDa smaller than their native source (Fig. 4c), indicating extensive post-translational modifications. Glycosylation of CelB and CelC was demonstrated using a periodic acid–Schiff base stain (Supplementary Fig. 10)^27^. CelA was not glycosylated. Interestingly, *C. bescii* CelA has been shown to have extensive glycosylation, further distinguishing the two proteins^28^. CelB is the most abundant protein in the complexes, but had low activities on CMC, PASC and Avicel (68.7 ± 27.9, 26.3 ± 0.1 and 0.9 ± 0.0 mU mg^–1^), consistent with the results obtained for the complex-bound CelB by zymography. CelC had the highest activities of the three proteins on CMC (2.06 ± 0.11 U mg^–1^) and PASC (0.19 ± 0.01 U mg^–1^), but had slightly lower activity on Avicel (32.0 ± 0.1 mU mg^–1^) compared to CelA (40.9 ± 0.8 mU mg^–1^). CelA had lower activities on PASC (67.1 ± 1.6 mU mg^–1^) and CMC (1.1 ± 0.1 U mg^–1^).

The CelABC complexes are distinct from cellulosomes isolated from anaerobic Firmicutes, because the cellulases in the CelABC complex are multidomain proteins containing carbohydrate-binding modules rather than dockerin domains^8^. Also absent in CelABC complexes is a non-catalytic scaffoldin protein containing the cohesin domains that bind the individual GHs. Glycosylation analysis of the complex revealed that it mainly contains O-linked galactooligosaccharides units (2–6 monomers) that are arranged in predominantly 1,2-glycosyl linkages (Supplementary Table 11 and Supplementary Fig. 11). The identification of O-glycosylation in CelB and CelC is consistent with the presence of Ser-Pro-Thr-rich linkers in both proteins (Supplementary Fig. 12). These linkers are often glycosylated in multidomain cellulases and in the scaffoldin protein CipA^29,30^. Although CelA also contains these linkers in its sequence, it was not glycosylated. The contribution of these O-linked galactooligosaccharides to the formation and stability of the CelABC complex was demonstrated by comparison of the proteolytic stability of the complex compared to *E*. coli-produced CelA and CelC, which form the complex with the highest molecular weight. The CelABC complexes were stable to Proteinase K treatment for 60 min, while both CelC and CelA were proteolysed (Supplementary Fig. 13).

## Discussion

This work has demonstrated that an aerobic cellulolytic microbial consortium derived from compost can be reproducibly cultivated and can be scaled to grow at 300 l. Therefore, this community provides an excellent model with which to understand the community dynamics of cellulolytic consortia and uncover enzymes missed by analysis focused solely on culturable isolates. Detailed study of the dynamics of the community using metagenomic methods established a succession in the community that is consistent with the definition of endogenous heterotrophic succession^18^. These metagenomic studies provided the basis for biochemical studies that identified a class of cellulase complexes in which O-linked glycosylation was critical for complex formation and stability.

The observation of O-linked galactooligosaccharides in CelB and CelC provides a hypothetical mechanism for complex formation. Although O-linked glycosylation is widespread in proteins from bacterial pathogens, it has not been shown to be involved in complex formation for GHs^31^. We propose that the galactooligosaccharides are bound to the threonine and serine linkages in the protein, and these glycosylated linkers form interprotein interactions with the CBM3 domains present in each of the CelABC component proteins. CBM3s have two predicted carbohydrate-binding sites on opposite faces of the domain^32^. The first binding site is a planar array of aromatic and polar residues that are predicted to bind crystalline cellulose. The second binding site on the opposite face, which also has polar and aromatic residues, has a shallow groove whose function is unknown. A previous study demonstrated weak binding between the shallow groove of a CBM3 and a peptide representing a linker consensus sequence from CipA^33^. Glycosylation of these linkers may strengthen the interaction with the shallow groove of the CBM3, providing a mechanism for the formation of the CelABC complexes. Glycosylation-dependent formation of a protein complex has been observed for insulin growth factors (IGFs), in which N-linked glycans are required for an 85 kDa glycoprotein to form a ternary complex with the IGFs in human serum^34^.

The unusual stability of the CelABC complexes, as demonstrated by their presence at the end of the cultivation and their resistance to proteolysis, complicates a simple mechanistic description linking the succession observed in the microbial community with activity of the celluases. Initial CMCase activity measurements for the 15 l and 300 l cultures indicated that some residual activity remains at the beginning of the culture (Fig. 1b and Supplementary Fig. 1b). This residual activity may be responsible for initial hydrolysis of the crystalline cellulose independent of the enzyme produced during subsequent cultivation. During the 2 week cultivations, the CMCase activity increased ~4–7-fold in the cultures at approximately the same time as the relative abundances in the community shifted from the dominance of ‘*Ca*. R. cellulovorans’, suggesting that the majority of this cellulase activity is produced during cultivation. Therefore, the cellulase activities that may influence the composition of the consortium may arise from residual enzymes carried over from previous cultivations, as well as enzymes produced during each 2 week cultivation by ‘*Ca*. R. cellulovorans’. This interplay may be critical for maintaining the remarkable stability of the consortium, which has retained similar community membership and dynamics through multiyear serial cultivations and scaling by 6,000-fold. A second complication in mechanistic interpretation is that the nature of the cellulose substrate may change during the cultivation, so that the CelABC complexes may be more effective over time, independent of the influence of the ‘*Ca*. R. cellulovorans’ population that initially produced them. Evidence for this phenomenon has been observed in the cellulolytic consortium adapted from Milipitas, CA, compost microbiota described above^13^, in which the residual cellulose becomes decrystallized during the 2 week cultivation. The difficulty in distinguishing the effects of microbial and enzymatic deconstruction and accounting for the dynamic nature of the cellulose substrate during cultivation emphasizes that the mechanisms underlying the proposed endogenous heterotrophic succession may be more complicated than a simple microbial succession.

The activity of the CelABC complexes on crystalline cellulose is sufficient (0.38 mmol h^−1^ l^−1^) to hydrolyse ~15% of the cellulose in the 300 l cultivation. Therefore, the complexes may serve as public goods^14^, releasing soluble glucans that support the other community members over the 2 week cultivation. A similar provision of GHs as public goods has been observed in defined co-cultures of human gut Bacteroidales isolates, in which species that generate extracellular GHs (producers) support the growth of species that lack these genes (recipients), and the recipients outcompete the producers when the growth of the producer is limited^35^. Interestingly, the inferred recipients in this cellulolytic consortium, *T. bispora* and *R. marinus*, generate substantial amounts of extracellular cellulases under other cultivation conditions^36,37^, but are not the dominant cellulase producers in the community cultivations described here, which suggests that glucans released by the CelABC complexes may repress subsequent synthesis of cellulases from other community members.

This work demonstrates that adapted consortia can provide microbial community models that can be used to begin to understand the interplay of enzymes and microbes in the deconstruction of plant biomass by microbial consortia. Metagenomic studies of complex microbial communities that hydrolyse plant polysaccharides contain highly abundant populations with genomes that have large numbers of  GH genes^38,39^. These abundant populations are often assigned as the primary GH producers in the environments they inhabit. The results described here suggest that transient community members in these complex consortia may produce highly active and stable extracellular GHs that are active independently of the populations that produce them and can act as public goods for the community.

## Methods

### Sample collection and enrichment of thermophilic consortia

The sample collection and enrichment procedures have been described previously^15^. Briefly, compost samples were collected from Jepson Prairie (JP) Organics, located in Vacaville, CA, in 2008. The compost-derived microbial consortium was initially grown aerobically with unpretreated switchgrass and then switched to grow on microcrystalline cellulose (1% wt/vol; Sigma) as the sole carbon source in liquid M9 medium augmented with vitamins. The enrichments were grown at 60 °C and 200 r.p.m. under aerobic conditions in an aerial rotary shaker and serially passed every 14 days with 4% vol/vol inoculum, referred to as passages. Cultivation of the 50 ml culture after passage 80 was scaled at the Advanced Biofuels Process Demonstration Unit, Lawrence Berkeley National Laboratory. A 500 ml culture was inoculated with a 2.5% vol/vol sample from the 50 ml culture and incubated at 60 °C for 14 days at 150 r.p.m. on a rotary shaker. This culture was inoculated into a 19 l bioreactor (Bioengineering USA) to a total volume of 15 l. The 15 l culture was grown at 60 °C, 150 r.p.m. and 0.26 volume gas (sterile air) per volume liquid per minute (VVM) for 14 days. A 400 l bioreactor (ABEC) was inoculated with 7.5 l of culture from the 19 l bioreactor to a volume of 300 l and incubated for 14 days at 60 °C, 150 r.p.m. agitation and 0.25 VVM air sparging. After 14 days, the final fermentation broth was centrifuged using an Alfa Laval Disc Stack centrifuge at 9,000*g* at 100 l h^–1^ with 30 min cell discharge interval and 125 kPa back pressure. The clarified broth was collected in the 300 l holding tank bioreactor and the pelleted biomass collected and stored at –80 °C. The supernatant was concentrated through a tangential flow filtration system with a 10 kDa Biomax filter membrane (EMD Millipore). The transmembrane pressure was set at 13 p.s.i. with feed pressure of 30 p.s.i. The concentrated supernatant was freeze-dried under vacuum for 24 h using a lyophilizer (Labconco) and the resulting powder was stored at –20 °C. CMCase and xylanase activities were measured daily for the 15 l and 300 l bioreactor cultivations by removal of samples each day from the bioreactors and the supernatant assayed as described below.

#### Sequencing, assembly and binning of metagenomics reads

DNA purification from samples extracted from the 15 l and 300 l bioreactors was performed as previously described^13^. Illumina sequencing (250 bp × 2) of the metagenomic samples was carried out by the Joint Genome Institute (JGI) and performed as previously described^40^. The sequencing reads of the DNA samples recovered from the 15 l bioreactor (days 1–14 were trimmed using Trimmomatics (with parameter ILLUMINACLIP:TruSeq3-PE.fa:2:30:10 LEADING:3 TRAILING:3 SLIDINGWINDOW:4:15 MINLEN:36)^41^ and co-assembled using IDBA-UD^42^ with the --pre-correction parameter. The 300 l samples (days 4, 5, 7, 10, 12 and 14) were also co-assembled, using the same settings as the 15 l samples. The co-assembled samples for the 15 l and 300 l bioreactor experiments were then binned using MaxBin 2.0^43^ with default parameters, yielding population genomes. The completeness and contamination ratios of these population genomes were assessed using CheckM^44^. Genomes with >10% of contamination rates were re-binned using MaxBin 2.0 by setting the input contig to the genome file and the input abundance to the extracted abundance files during the whole metagenome binning. The output bins were re-examined using CheckM and the produced bins with higher completeness were chosen to replace the original genome, while the other bins with higher levels of contaminants were discarded. The most likely taxonomic ranks of the recovered genomes were predicted by searching the predicted proteins against the NCBI non-redundant (NR) database, collecting and processing the hits using the least common ancestor (LCA) algorithm proposed by MEGAN4^45^ and assigning the most probable taxonomic rank to the recovered genomes according to the LCA results. GH genes present in the recovered population genomes were identified by using Prodigal^46^ and then annotated using dbCAN^47^.

DNA was isolated from a 50 ml aerobic shake flask culture with a bacterial consortium that was adapted from green waste compost obtained from Newby Island Sanitary Landfill in Milpitas, CA, by growth on crystalline cellulose. The enzymatic activities and microbial community membership of this consortium have been described previously^13^. Metagenomic sequencing was performed by the JGI as described above, and the sequenced reads were assembled as previously described^40^. Population genomes were recovered by automated binning with Maxbin^43^ and checked for completeness and contamination with CheckM^44^.

### 16S and 23S rRNA gene analysis

A partial 16S rRNA gene (706 bp) was recovered from the ‘*Ca*. R. cellulovorans’ metagenomic bin. This fragment was used to identify a nearly full-length 16S rRNA gene (1,597 bp; 99.7% identical JGI gene ID Ga0074251_1085371) that was recovered from the initial assembled metagenome (JGI taxon ID 3300005442) obtained for this consortium when it had been adapted to switchgrass^40^. This observation indicated that ‘*Ca*. R. cellulovorans’ was present in the consortium when it was adapted to switchgrass, before transferring the consortium to grow on microcrystalline cellulose. Sequences from three clones (GenBank accessions KC978751, KC978760 and KC978763) were >99% identical to portions of the full-length rRNA ‘*Ca*. R. cellulovorans’ sequence. These clones were recovered from DNA samples isolated from a time series (14 days) of an adaptation of compost from Newby Island Landfill (Milipitas, CA, USA) to grow with microcrystalline cellulose as the sole carbon source at 60 °C^13^. As described above, a partial genome was recovered from this cultivation that was >99% identical at the amino acid level to the ‘*Ca*. R. cellulovorans’. A partial 23S rRNA gene (1,444 bp) was recovered from the ‘*Ca*. R. cellulovorans’ metagenomic bin. The 16S and 23S rRNA gene sequences were aligned using MUSCLE^48^ trimmed using Gblocks^49^, and the phylogenetic tree was constructed using MEGA5^50^ with the Tamura–Nei model. Bootstrap values were calculated with 1,000 replicates.

### Reconstruction of ‘*Ca*. R. cellulovorans’ GH gene cluster

Six small contigs were identified in the ‘*Ca*. R. cellulovorans’ population genome, which contained partial genes containing a catalytic domain (GH9, GH48, GH6/5, 2×GH10, AA10) linked to at least one CBM3. The clustering of these genes was confirmed by PCR amplification of DNA isolated from the cellulolytic consortium. PCR primers (Supplementary Table 8) were designed using the CLC Main Workbench (Qiagen) and PCR products were cloned into pJET1.2/blunt Cloning Vector (Fermentas) and sequenced with an ABI system according to the manufacturer’s instructions. Assembly of gene sequences into a gene cluster and annotation of genes was performed with the CLC Main Workbench and checked for chimerae using the Bellerophon algorithm^51^.

### Phylogenetic analysis

Alignments of protein sequences were performed using the CLUSTALW multiple alignment accessory application in the CLC Main Workbench (Qiagen). In brief, phylogenetic trees were constructed using the CLC Main Workbench applying the maximum likelihood method based on the Whelan and Goldman protein substitution model^52^. Bootstrap values were calculated with 1,000 replicates.

To build the concatenated protein tree, genes were first searched against the PFAM profiles^53^ using HMMER3^54^. Genes with PFAM annotations that appear once and only once across all involved genomes were aligned separately using MUSCLE^48^. After the alignments were concatenated and trimmed using Gblocks^49^, the concatenated maximum-likelihood protein tree was constructed using MEGA5^50^ with the JTT (Jones, Taylor, Thorton) model. Bootstrap values were estimated with 1,000 replicates.

### Protein purification

Lyophilized supernatant (170 mg) obtained from the 300 l cultivation was dissolved in 5 ml H_2_O and passed through a 0.2 µm filter. The supernatant was desalted by dialysis against the buffer (20 mM Tris, pH 8.0) for 24 h with three buffer changes, followed by a 30 ml NaCl gradient fractionation (0–2 M NaCl) using a 5 ml HiTrap Q HP column on an ÄKTA Protein Purification System (GE Healthcare).

Cellulases in the supernatant from the 300 l cultivation were also enriched by binding to phosphoric acid swollen cellulose (PASC), an adaptation of a procedure previously described for cellulosome purification from *Clostridium thermocellum* ^26^. Briefly, 250 mg of lyophilized PASC produced from Avicel PH-105 was added to 500 mg of supernatant dissolved in 10 ml H_2_O and mixed at room temperature with a magnetic stir bar for 30 min. After a binding step at 4 °C for 2 h, the amorphous cellulose was centrifuged for 10 min at 3,000*g* and rigorously washed for 6 cycles with 25 ml reaction buffer (25 mM 2-(*N*-morpholino)ethanesulfonic acid (MES), pH 6.0). Washed PASC was resuspended in 10 ml reaction buffer and transferred into dialysis membranes (SnakeSkin and Slide-A-Lyzer; Fisher Scientific) with a 3.5–10 kDa cutoff and dialysed at 60 °C against 4 l reaction buffer at 55–60 °C for up to 48 h with three buffer exchanges per day to prevent possible product inhibition. Dialysis membranes used in this study consisted of regenerated cellulose and were destabilized by cellulases of the substrate, and thus needed to be exchanged every 24 h to prevent membrane rupture. The reaction was considered complete after no visible changes to the substrate were observable. By centrifugation for 20 min at 3,000*g* the enrichment was split into residual biomass (in the pellet) and the affinity digestion protein fraction (in the supernatant, AD).

### Measurement of protein concentration and GH activity

Protein concentrations were determined using the bicinchoninic (BCA) assay (Pierce BCA Protein Assay Kit, Thermo Scientific) method using a 96-well plate (200 μl reaction volume) with bovine serum albumin as the standard. CMCase and xylanase activity assays were conducted as described previously^55^. Enzyme activity units (U) were defined as µmol of sugar liberated per min. Enzyme activity units for supernatant preparations were calculated as U per ml of supernatant volume. CMC activity units of purified heterologously expressed proteins were reported as U per mg, representing specific activity measurements.

Soluble substrates (*p*-nitrophenyl (pNP)-labelled) with cellobiohydrolase (pNPC), β-d-dglucosidase (pNPG), β-D-xylosidase (pNPX) and α-l-arabinofuranosidase (pNPA) activities were used to determine enzyme activities on their respective substituents^56^. The *p*-nitrophenyl substrate (90 μl) was incubated with 10 μl of diluted enzyme, incubated for 30 min, and quenched with 50 μl of 2% cold sodium bicarbonate. The absorbance of released *p*-nitrophenyl was measured at 410 nm. Activities using *p*-nitrophenyl substrates were calculated as U ml^−1^.

### Saccharification of cellulose substrates

Saccharifications were performed in the presence of 2% (wt/vol) Avicel (Sigma) and PASC. Each mixture was prepared in 50 mM MES, pH 6.0 with 10 mg protein per g glucan in biomass to a final volume of 625 µl in a 2 ml screw-cap vial. Saccharifications were carried out at 70 °C in a shaker for 72 h, with 50 µl samples taken every 24 h. All hydrolysates were collected via centrifugation at 21,000*g* for 5 min and 0.45 μm filtered to remove large biomass particles prior to sugar analysis. After filtration, samples were kept frozen at −20 °C and thawed before analysis. Glucose concentrations were measured on an Agilent 1200 Series HPLC system equipped with an Aminex HPX-87H column (Bio-Rad) and refractive index detector. Samples were run with an isocratic 4 mM sulfuric acid mobile phase. Sugar concentrations were determined using standards containing cellotriose, cellobiose, glucose, xylose and arabinose.

### PAGE and zymograms

SDS–PAGE was performed with 8–16% Protean TGX protein gradient gels (Bio-Rad) with the Tris-glycine-SDS buffer^57^. Blue Native (BN)–PAGE^58^ was performed with 3–12% NativePAGE Bis-Tris protein gradient gels (Thermo Scientific) in presence of 0.02% Coomassie Blue G-250. For subunit analysis of native complexes, individual lanes from the BN–PAGE were excised, incubated in 2% SDS and 160 mM dithiothreitol (DTT), and denatured at 95 °C for 10 min, unless otherwise indictated (Fig. 3e). Proteins were separated with 8% polyacrylamide gels, which were hand cast. Protein bands were stained with SimplyBlue SafeStain Coomassie Blue dye (Thermo Scientific) according to the manufacturer’s instructions.

Protein bands with activity on CMC and xylan were visualized using modification of the zymogram technique, as described previously^15^. Gels were incubated in 2% wt/vol CMC or 2% wt/vol birchwood xylan solutions followed by incubation at 60 °C for up to 2 h in reaction buffer (25 mM MES, pH 6.0). In-gel enzymatic activities were visualized by incubating gels with a 0.5% Congo Red solution for 15 min and subsequent multiple washing steps with 20% NaCl.

### Glycosylation analysis

Protein glycosylations were visualized in-gel by the periodic acidic Schiff stain^27^ using a Pierce Glycoprotein Staining Kit (Thermo Scientific) according to the manufacturer’s instructions.

N-glycan analysis was performed as described previously^59^. However, no N-linked glycans were detected. Total glycosyl compositional analysis was performed by combined gas chromatography/mass spectrometry (GC/MS) of the per-O-trimethylsilyl (TMS) derivatives of the monosaccharide methyl glycosides produced from the sample by acidic methanolysis^60^. O-linked glycans were released by β-elimination and permethylated^59^. The permethylated O-linked glycans were analysed by matrix assisted laser desorption/ionization-time of flight (MALDI–TOF) and electrospray ionization tandem mass spectrometry (ESI MS/MS)^61^ and gas chromatography/mass spectrometry (GC/MS) for linkage analysis^62^.

### Proteinase K digestion

The *E. coli*-expressed CelA and CelC and the AD fraction were digested at 50 °C for 60 min in reaction buffer (20 mM Tris-HCl, 400 mM NaCl and 0.3% SDS, 5 mM EDTA containing 75 µg of respective enzyme and 3.75 µg proteinase K). After heat inactivation of proteinase K at 95 °C, the reaction mixture was analysed by SDS–PAGE (8–16% gradient).

### Proteomic analysis

Proteins were digested from SDS–PAGE gels as previously described^63^. Samples were analysed on an Agilent 6550 iFunnel QTOF mass spectrometer coupled to an Agilent 1290 UHPLC system, as described in ref. ^64^. Briefly, peptides were loaded onto an Ascentis Express Peptide ES-C18 column (10 cm length × 2.1 mm internal diameter, 2.7 µm particle size; Sigma Aldrich) operating at 60 °C and at a flow rate of 400 µl min^–1^. A 13.5 min chromatography method with the following gradient was used: the initial starting condition (95% Buffer A (0.1% formic acid) and 5% Buffer B (99.9% acetonitrile, 0.1% formic acid)) was held for 1 min. Buffer B was then increased to 35% in 5.5 min, followed by an increase to 80% B in 1 min, where it was held at 600 µl min^–1^ for 3.5 min. Buffer B was decreased to 5% over 0.5 min, where it was held for 2 min at 400 µl min^–1^ to re-equilibrate the column with the starting conditions. Peptides were introduced into the mass spectrometer from the UHPLC by using a Dual Agilent Jet Stream Electrospray Ionization source operating in positive-ion mode. The source parameters used include a gas temperature of 250 °C, drying gas at 14 l min^–1^, nebulizer at 35 p.s.i.g, sheath gas temp of 250 °C, sheath gas flow of 11 l min^–1^), *V*_Cap_ of 5,000 V, fragmentor *V* of 180 V and OCT(octopole) 1 RF (radio frequency) *V*_pp_ of 750 V. The data were acquired with Agilent MassHunter Workstation Software, LC/MS Data Acquisition B.06.01 (Build 6.01.6157). The resultant data files were searched against a data set containing reconstructed population genomes from the 300 l bioreactor, with common contaminants appended, with Mascot version 2.3.02 (Matrix Science), then filtered and refined using Scaffold version 4.6.1 (Proteome Software).

### Heterologous protein expression

Constructs for the CelABC genes were obtained both by PCR  amplification from metagenomic DNA with specific primers (Supplementary Table 8) and synthesis of codon-optimized versions for expression in *E. coli* (Gen9). Genes were cloned into the modified bacterial expression vector pET39b(+) vector with a T7/lac promoter and a TEV-cleavable C-terminal 6xHis tag but lacking the DsbA secretion sequence (Novagen) using Gibson assembly^65^. All reagents were purchased from New England Biolabs. The desired genes without their signal sequences and the expression vector were PCR-amplified, DpnI-digested and incubated with 1× Gibson assembly Master Mix for 15 min at 50 °C. The product was then transformed into chemically competent *E. coli* DH10α cells for storage and for heterologous protein expression into chemically competent *E. coli* BL21 (DE3). Starter cultures (50 ml) of *E. coli* BL21 (DE3) harbouring plasmids were grown overnight in LB medium containing 25 μg ml^–1^ kanamycin at 37 °C and shaken at 200 r.p.m. in rotary shakers. Expression was performed in Terrific broth with 2% glycerol, 25 μg ml^–1^ kanamycin and 2 mM MgSO_4_. Starter cultures were used to inoculate 1 l of expression medium in a 2 l baffled Erlenmeyer flask and incubated at 18 °C while shaking (200 r.p.m.), and induced with 500 µM isopropyl β-d-thiogalactopyranoside (IPTG). Following induction, cultures were again incubated at 18 °C. At 22 h, cultures were centrifuged at 15,500*g* for 30 min. Cell pellets were resuspended in 25 ml lysis buffer (50 mM NaPO_4_, 300 mM NaCl, 5 mM imidazole; pH 7.4) and homogenized with an EmulsiFlex-C3 instrument (Avestein). After incubation at 60 °C for 30 min, lysates were collected via centrifugation at 75,000*g* for 30 min and 0.45 μm filtered to remove large particles before purification. Polyhistidine-tagged proteins were purified on Cobalt-NTA resin (Thermo Scientific). To cleave the 6xHis Tag, 1 g purified protein was incubated with 50 mg His-tagged TEV-protease and simultaneously dialysed against 4 l reaction buffer (50 mM NaPO_4_, 300 mM NaCl; pH 7.4) for 24 h and three reaction buffer exchanges. After a second purification step via Cobalt-NTA resin, the flow-through fractions contained the purified and untagged proteins. Proteins were stored at 4 °C until ready for use. The proteins were >90% pure as visualized by SDS–PAGE (Fig. 4c).

### Life Sciences Reporting Summary

Further information on experimental design and reagents is available in the Life Sciences Reporting Summary.

### Data availability

Metagenomic sequencing data can be accessed at the JGI IMG website (http://img.jgi.doe.gov/) or the JGI Genome Portal (http://genome.jgi.doe.gov/), and the specific IMG genome IDs are listed in Supplementary Table 12. The draft genome sequence for ‘*Candidatus* Reconcilbacillus cellulovorans’ has been deposited at GenBank (MOXJ00000000). The gene sequences and plasmid constructs for the ‘*Ca*. Reconcilbacillus’ cellulases CelA (JPUB_007824), CelB (JPUB_007826) and CelC (JPUB_007828) are available from the public version of the JBEI Registry (https://public-registry.jbei.org) and are physically available from the authors and/or Addgene (http://www.addgene.org) upon request.

## Electronic supplementary material

Supplementary Information Supplementary Tables 1–12 and Supplementary Figures 1–17.

Life Sciences Reporting Summary
