# Supplementary material for: A bacterial pioneer produces cellulase complexes that persist through community succession
Source: Nat Microbiol. 2017 Nov 6;3(1):99–107. doi: 10.1038/s41564-017-0052-z (PMC6794216; doi:10.1038/s41564-017-0052-z)

nature microbiology

SUPPLEMENTARY INFORMATION

https://doi.org/10.1038/s41564-017-0052-z

In the format provided by the authors and unedited.

A bacterial pioneer produces cellulase complexes that persist through community succession

Sebastian Kolinko[1,2,11], Yu-Wei Wu[1,2,3], Firehiwot Tachea[2,4], Evelyn Denzel[1,5,6], Jennifer Hiras[1,5,12], Raphael Gabriel[1,2,7], Nora Bäcker[1,5,6], Leanne Jade G. Chan[1,2], Stephanie A. Eichorst[1,5,13], Dario Frey[1,2,6], Qiushi Chen[8], Parastoo Azadi[8], Paul D. Adams[1,9], Todd R. Pray[2,4], Deepti Tanjore[2,4], Christopher J. Petzold[1,2], John M. Gladden[1,10], Blake A. Simmons[1,2] and Steven W. Singer[1,2]*

[1]Joint BioEnergy Institute, Emeryville, CA, USA. [2]Biological Systems and Engineering Division, Lawrence Berkeley National Laboratory, Berkeley, CA, USA. [3]Graduate Institute of Biomedical Informatics, College of Medical Science and Technology, Taipei Medical University, Taipei, Taiwan. [4]Advanced Biofuels Process Development Unit, Lawrence Berkeley National Laboratory, Emeryville, CA, USA. [5]Physical Biosciences Division, Lawrence Berkeley National Laboratory, Berkeley, CA, USA. [6]Faculty of Biotechnology, University of Applied Sciences, Mannheim, Germany. [7]Institut für Genetik, Technische Universität Braunschweig, Braunschweig, Germany. [8]Complex Carbohydrate Research Center, University of Georgia, Athens, GA, USA. [9]Molecular Biophysics and Integrated Bioimaging Division, Lawrence Berkeley National Laboratory, Berkeley, CA, USA. [10]Biological and Materials Science Center, Sandia National Laboratories, Livermore, CA, USA. Present addresses: [11]Department of Chemistry, University of Basel, Basel, Switzerland. [12]Corning Incorporated, Corning, NY, USA. [13]Division of Microbial Ecology, Department of Microbiology and Ecosystem Science, Research network "Chemistry meets Microbiology", University of Vienna, Vienna, Austria. *e-mail: swsinger@lbl.gov

© 2017 Macmillan Publishers Limited, part of Springer Nature. All rights reserved.

Supplementary Tables

Supplementary Table 1. Genome information of the bins extracted from the 15 L cultivation.

Bin	Taxonomy	Closest species (amino acid identity)	Genome size	GC %	Completeness[1]	Contamination[2]
001	*Rhodothermus marinus*	*Rhodothermus marinus* (98.32%)	3115637	64.6	73.8%	12.27%
002	*Thermus thermophilus*	*Thermus thermophilus* (96.59%)	1985524	68.4	71.0%	6.93%
003	*Thermobispora bispora*	*Thermobispora bispora* (98.95%)	4383805	72.1	98.1%	5.31%
004	*Thermobacillus*	*Thermobacillus composti* (87.80%)	4410654	62.7	100%	0.44%
005	*Gemmatimonadetes*	*Gemmatimonadetes bacterium* KBS708 (59.68%)	3786842	71.4	93.5%	5.43%
006	*Geobacillus*	*Geobacillus* sp. JF8 (89.53%)	3332863	51.4	97.2%	2.47%
007	*Paenibacillaceae* 2	*Cohnella laeviribosi* (72.38%)	3650356	60.9	99.1%	2.36%
**008**	***Paenibacillaceae* 1**	***Paenibacillus ginsengihumi* (65.98%)**	**2917013**	**60.0**	**96.3%**	**1.34%**
009	*Paenibacillaceae* 3	*Cohnella laeviribosi* (70.07%)	3667478	62.1	98.1%	3.01%
010	*Solirubrobacterales*	*Conexibacter woesei* (63.45%)	2377682	67.6	86.0%	2.01%
011	*Alphaproteobacteria*	*Filomicrobium* sp. W (67.38%)	3530889	60.5	92.5%	2.78%
012	*Sphaerobacter thermophiles*	*Sphaerobacter thermophilus* (93.65%)	4275950	68.3	93.5%	1.71%
013	*Alphaproteobacteria*	*Filomicrobium* sp. W (67.20%)	3663535	61.1	95.3%	0.87%
014	*Thermomicrobium*	*Thermomicrobium roseum (80.69%)*	2256146	65.2	85.0%	1.35%
015	*Thermobaculum terrenum*	*Thermobaculum terrenum* (98.07%)	3115566	52.2	88.8%	5.95%
016	*Rubrobacter xylanophilus*	*Rubrobacter xylanophilus* (99.10%)	3813100	70.2	96.3%	7.66%
017	*Bacillaceae*	*Thermobacillus composti* (74.89%)	4961510	57.0	99.1%	1.06%
018	*Ralstonia pickettii*	*Ralstonia pickettii* (96.18%)	5436724	63.5	95.3%	7.68%
019	*Sphaerobacter thermophiles*	*Sphaerobacter thermophilus* (98.67%)	813062	65.7	5.6%	2.59%
020	*Bacillaceae*	*Paenibacillus ginsengihumi* (69.46%)	3121668	55.4	94.4%	0.87%
021	*Paenibacillaceae* 4	*Thermobacillus composti* (72.34%)	2899243	63.5	99.1%	1.27%

[1] Completeness was measured based on 107 marker genes.

[2] Contamination ratios were estimated using CheckM.

Supplementary Table 2. Average genome coverages for the 15L metagenomes.

Bin	Day01	Day02	Day03	Day04	Day05	Day06	Day07	Day08	Day09	Day10	Day11	Day13	Day14
001	3422.28	997.26	897.72	499.2	730.71	803.27	654	684.65	725.89	908.79	675.22	605.16	1125.18
002	1229.4	427.58	434.33	334.7	394.81	379.03	259.79	243.73	261.88	303.13	256.37	351.79	720.82
003	379.83	100.47	81.63	98.99	250.46	215.04	387.53	361.69	478.09	656	517.04	355.73	356.28
004	317.43	64.41	72.36	33	15.2	27.78	20.09	12.45	8.94	6.62	2.74	1.44	1.64
005	257.98	79.41	58.19	47.18	47.02	68.59	91.61	95.7	100.07	158.03	115.48	105.05	183.02
006	270.63	35.96	12.54	2.36	1.51	1.11	1.22	0.8	0.75	0.79	0.55	0.46	0.42
007	138.44	77.78	227.7	543.31	309.74	77.25	55.01	39.91	33.56	32.5	22.77	29.27	37.31
**008**	**84.55**	**81.37**	**685.04**	**1086.37**	**595.74**	**111.55**	**83.36**	**67.08**	**50.38**	**39.47**	**22.31**	**24.86**	**33.17**
009	27.01	12.27	15.38	23.3	24.82	16.84	10.45	7.97	7.57	12.57	10.54	14.17	23.7
010	38.19	8.53	4.82	1.12	1.18	0.58	0.7	0.57	0.65	1.66	1.72	3.07	1.74
011	26.62	7.01	6.31	6.48	13.08	16.99	18.14	14.17	11.74	14.14	8.15	5.01	2.7
012	19.51	5.52	3.84	1.74	2.66	4.62	5.83	6.23	7.38	12.15	9.4	13.01	9.66
013	14.16	3.63	3.98	3.37	5.74	6.34	7.91	7.08	8.69	13.24	8.71	8.33	7.52
014	14.75	4.11	2.92	1.49	1.85	2.34	2.71	3.03	3.68	7.08	5.09	8.22	5.16
015	8.33	2.01	0.93	0.45	0.74	0.93	0.84	1.03	1.35	2.76	2.88	6.77	5.55
016	7.01	4.63	4.49	1.78	0.58	0.96	1.68	1.52	2.38	4.72	3.61	4.4	2.09
017	2.28	0.33	0.92	8.29	0.69	2.94	1.98	1.06	0.94	1.04	0.7	1.09	2.08
018	2.1	0.41	0.15	0.35	0.09	0.42	0.53	0.48	0.41	0.07	0.57	0.56	0.68
019	0.99	0.32	0.28	0.15	0.12	0.19	0.28	0.24	0.29	0.54	0.37	0.5	0.36
020	1.16	1.63	1.06	0.42	0.31	0.23	0.32	0.27	0.38	0.88	0.72	0.61	1
021	0.3	0.19	0.48	1.97	1.64	0.98	0.83	0.59	0.51	0.47	0.3	0.24	0.34

Supplementary Table 3. Genome information of the bins extracted from the 300 L cultivation.

Bin	Taxonomy	Closest species (amino acid identity)	Genome size	GC %	Completeness[1]	Contamination[2]
**001**	***Paenibacillaceae* 1**	***Paenibacillus ginsengihumi* (66.06%)**	**2975368**	**60.1**	**96.3%**	**1.63%**
002	*Rhodothermus marinus*	*Rhodothermus marinus* (98.34%)	2650283	64.3	72.9%	7.03%
003	*Thermus thermophilus*	*Thermus thermophilus* (96.38%)	2232390	68.8	84.1%	2.67%
004	*Paenibacillaceae* 2	*Cohnella laeviribosi* (70.65%)	3292257	61.1	99.1%	0.52%
005	*Thermobacillus*	*Thermobacillus composti* (87.66%)	4307311	62.7	100%	0.09%
006	*Paenibacillaceae* 3	*Cohnella laeviribosi* (70.01%)	3728631	61.9	97.2%	0.87%
007	*Thermobispora bispora*	*Thermobispora bispora* (98.87%)	4874851	71.2	98.1%	5.10%
008	*Gemmatimonadetes*	*Gemmatimonadetes bacterium* KBS708 (59.68%)	3796215	71.3	95.3%	5.43%
009	*Bacillaceae*	*Thermobacillus composti* (73.97%)	4791899	57.2	99.1%	4.23%
010	*Alphaproteobacteria*	*Filomicrobium sp. W* (66.89%)	3723463	61.2	99.1%	0%
011	*Thermomicrobia*	*Thermomicrobium roseum* (80.60%)	2358714	65.1	87.9%	3.84%
012	*Sphaerobacter thermophiles*	*Sphaerobacter thermophilus* (93.33%)	4739352	68	93.5%	9.81%
013	*Rubrobacter xylanophilus*	*Rubrobacter xylanophilus* (99.07%)	4127530	69.4	96.3%	6.35%
014	*Geobacillus*	*Geobacillus* sp. JF8 (89.43%)	1394897	52.5	20.6%	0.16%
015	*Thermobaculum terrenum*	*Thermobaculum terrenum* (98.47%)	2028580	48.4	82.2%	0%
016	*Solirubrobacterales*	*Conexibacter woesei* (63.76%)	2003368	70.4	72.0%	3.39%
017	*Ralstonia pickettii*	*Ralstonia pickettii* (96.15%)	3276067	63.6	61.7%	2.94%

[1] Completeness was measured based on 107 marker genes.
[2] Contamination ratios were estimated using CheckM.

Supplementary Table 4. Average genome coverages for the 300L metagenomes.

Bin	Day04	Day05	Day07	Day10	Day12	Day14
001	1299.04	653.75	93.22	28.54	11.29	13.17
002	508.7	773.91	1147.41	1270.15	1249.27	830.8
003	370.85	179.36	180.84	83.85	76.77	89.28
004	217.92	112.03	25.65	15.71	7.98	10.11
005	114.97	124.8	29.69	6.42	4.18	3.64
006	32.7	47.79	16.18	9.86	6.87	17.52
007	29.74	110.47	249.05	219.93	110.01	361.43
008	13.19	26.71	45.5	81.3	54.97	162.48
009	9.77	12.2	5.7	1.85	2.12	3.46
010	3.63	11.6	26.04	19.49	11.95	38.6
011	1.95	2.68	9.8	5.85	7.95	53.1
012	0.78	1.25	2.67	1.92	2.1	9.41
013	0.62	0.56	1.15	0.39	0.41	10.65
014	0.5	0.58	0.48	0.27	0.32	1.96
015	0.25	0.52	1.18	0.58	1.27	9.86
016	0.19	0.18	0.17	0.11	0.09	6.37
017	0.12	1	0.84	0.58	1.03	1.02

Supplementary Table 5. Genome features of *Candidatus* ‘Reconcilibacillus cellulovorans’.

**Genome size**	2,922,051 bp
**Scaffold number**	114
**Maximum scaffold length**	266,707
**N50 scaffold length**	70,320
**G+C content**	60
**Number of predicted CDS**	2814
**Number of RNAs**[1]	
Number of 5S rRNA	2
Number of 16S rRNA	3
Number of 23S rRNA	3
Number of tRNA	47
**Estimated completeness**	96.3%
Total marker gene number	105
Unique marker gene number	103

[1]Including rRNA fragments

Supplementary Table 6. The 86 shared marker genes that were used to build the phylogenetic tree (Figure 2B)

Pfam domain	Pfam name	Pfam description
PF00886.14	Ribosomal_S16	Ribosomal protein S16
PF14622.1	Ribonucleas_3_3	Ribonuclease-III-like
PF08529.6	NusA_N	NusA N-terminal domain
PF00406.17	ADK	Adenylate kinase
PF00318.15	Ribosomal_S2	Ribosomal protein S2
PF10396.4	TrmE_N	GTP-binding protein TrmE N-terminus
PF06071.8	YchF-GTPase_C	Protein of unknown function (DUF933)
PF03948.9	Ribosomal_L9_C	Ribosomal protein L9, C-terminal domain
PF03775.11	MinC_C	Septum formation inhibitor MinC, C-terminal domain
PF01245.15	Ribosomal_L19	Ribosomal protein L19
PF13331.1	DUF4093	Domain of unknown function (DUF4093)
PF01250.12	Ribosomal_S6	Ribosomal protein S6
PF04551.9	GcpE	GcpE protein
PF07949.7	YbbR	YbbR-like protein
PF00177.16	Ribosomal_S7	Ribosomal protein S7p/S5e
PF00312.17	Ribosomal_S15	Ribosomal protein S15
PF10635.4	DisA-linker	DisA bacterial checkpoint controller linker region
PF02580.11	Tyr_Deacylase	D-Tyr-tRNA(Tyr) deacylase
PF00217.14	ATP-gua_Ptrans	ATP:guanido phosphotransferase, C-terminal catalytic domain
PF08459.6	UvrC_HhH_N	UvrC Helix-hairpin-helix N-terminal
PF01715.12	IPPT	IPP transferase
PF05496.7	RuvB_N	Holliday junction DNA helicase ruvB N-terminus
PF00231.14	ATP-synt	ATP synthase
PF00466.15	Ribosomal_L10	Ribosomal protein L10
PF12344.3	UvrB	Ultra-violet resistance protein B
PF00410.14	Ribosomal_S8	Ribosomal protein S8
PF09547.5	Spore_IV_A	Stage IV sporulation protein A (spore_IV_A)
PF04079.11	DUF387	Putative transcriptional regulators (Ypuh-like)
PF01176.14	eIF-1a	Translation initiation factor 1A / IF-1
PF00673.16	Ribosomal_L5_C	ribosomal L5P family C-terminus
PF04468.7	PSP1	PSP1 C-terminal conserved region
PF09182.5	PuR_N	Bacterial purine repressor, N-terminal
PF00542.14	Ribosomal_L12	Ribosomal protein L7/L12 C-terminal domain
PF00572.13	Ribosomal_L13	Ribosomal protein L13
PF12116.3	SpoIIID	Stage III sporulation protein D
PF03419.8	Peptidase_U4	Sporulation factor SpoIIGA
PF01351.13	RNase_HII	Ribonuclease HII
PF00347.18	Ribosomal_L6	Ribosomal protein L6

PF03602.10	Cons_hypoth95	Conserved hypothetical protein 95
PF01632.14	Ribosomal_L35p	Ribosomal protein L35
PF00327.15	Ribosomal_L30	Ribosomal protein L30p/L7e
PF04026.7	SpoVG	SpoVG
PF00276.15	Ribosomal_L23	Ribosomal protein L23
PF03772.11	Competence	Competence protein
PF02033.13	RBFA	Ribosome-binding factor A
PF01509.13	TruB_N	TruB family pseudouridylate synthase (N terminal domain)
PF01649.13	Ribosomal_S20p	Ribosomal protein S20
PF00344.15	SecY	SecY translocase
PF00213.13	OSCP	ATP synthase delta (OSCP) subunit
PF00430.13	ATP-synt_B	ATP synthase B/B' CF(0)
PF04997.7	RNA_pol_Rpb1_1	RNA polymerase Rpb1, domain 1
PF00297.17	Ribosomal_L3	Ribosomal protein L3
PF01205.14	UPF0029	Uncharacterized protein family UPF0029
PF01016.14	Ribosomal_L27	Ribosomal L27 protein
PF01219.14	DAGK_prokar	Prokaryotic diacylglycerol kinase
PF05848.6	CtsR	Firmicute transcriptional repressor of class III stress genes (CtsR)
PF00828.14	Ribosomal_L18e	Ribosomal protein L18e/L15
PF00861.17	Ribosomal_L18p	Ribosomal L18p/L5e family
PF00829.16	Ribosomal_L21p	Ribosomal prokaryotic L21 protein
PF00687.16	Ribosomal_L1	Ribosomal protein L1p/L10e family
PF00189.15	Ribosomal_S3_C	Ribosomal protein S3, C-terminal domain
PF00338.17	Ribosomal_S10	Ribosomal protein S10p/S20e
PF01195.14	Pept_tRNA_hydro	Peptidyl-tRNA hydrolase
PF02527.10	GidB	rRNA small subunit methyltransferase G
PF00453.13	Ribosomal_L20	Ribosomal protein L20
PF00889.14	EF_TS	Elongation factor TS
PF00416.17	Ribosomal_S13	Ribosomal protein S13/S18
PF00366.15	Ribosomal_S17	Ribosomal protein S17
PF09551.5	Spore_II_R	Stage II sporulation protein R (spore_II_R)
PF03418.9	Peptidase_A25	Germination protease
PF01765.14	RRF	Ribosome recycling factor
PF02823.11	ATP-synt_DE_N	ATP synthase, Delta/Epsilon chain, beta-sandwich domain
PF00164.20	Ribosom_S12_S23	Ribosomal protein S12/S23
PF09546.5	Spore_III_AE	Stage III sporulation protein AE (spore_III_AE)
PF00831.18	Ribosomal_L29	Ribosomal L29 protein
PF01288.15	HPPK	7,8-dihydro-6-hydroxymethylpterin-pyrophosphokinase (HPPK)
PF01628.16	HrcA	HrcA protein C terminal domain
PF02130.12	UPF0054	Uncharacterized protein family UPF0054

PF02464.12	CinA	Competence-damaged protein
PF08769.6	Spo0A_C	Sporulation initiation factor Spo0A C terminal
PF00773.14	RNB	RNB domain
PF00380.14	Ribosomal_S9	Ribosomal protein S9/S16
PF03668.10	ATP_bind_2	P-loop ATPase protein family
PF03947.13	Ribosomal_L2_C	Ribosomal Proteins L2, C-terminal domain
PF01782.13	RimM	RimM N-terminal domain
PF03652.10	UPF0081	Uncharacterised protein family (UPF0081)

Supplementary Table 7. Genome information of the bins recovered from the Newby Island compost (NIC) microbiota adapted to grow on microcrystalline cellulose.

Bin	Taxonomy	Closest species	Genome size	GC %	Coverage	Completeness[1]	Contamination[2]
001	*Chitinophagaceae* NYFB	*Chitinophaga pinensis* (63.16%)	2875310	47	12905.55	93.5%	3.9%
002	*Thermus thermophilus*	*Thermus thermophilus* (96.13%)	2452761	67	1927.38	89.7%	7.21%
003	*Chlorobi bacterium* NICIL-2	*Chlorobi bacterium NICIL-2* (99.85%)	2813330	58	548.27	95.3%	0%
004	*Thermobacillus*	*Thermobacillus composti* (86.32%)	4135320	62	155.43	99.1%	4.46%
005	*Thermobacillus*	*Thermobacillus composti* (74.04%)	2981673	63	75.12	99.1%	5.57%
006	*Thermobispora bispora*	*Thermobispora bispora* (98.33%)	3364125	71	51.11	85.0%	16.62%
007	*Thermorudis peleae*	*Thermorudis peleae* (98.42%)	2910333	59	43.89	83.2%	9.35%
008	*Bacillaceae*	*Thermicanus aegyptius* (84.56%)	1554675	47	43.01	0.00%[3]	0%
009	*Verrucomicrobia*	*Pedosphaera parvula* (63.39%)	3167038	66	29.25	68.2%	7.6%
010	*Alphaproteobacteria*	*Filomicrobium* sp. W (67.29%)	3882381	60	24.56	78.5%	10.96%
**011**	**‘*Candidatus* Reconcillibacillus cellulovorans NIC’**	***Paenibacillus ginsengihumi* (67.86%)**	**1991453**	**60**	**24.13**	**74.8%**	**7.74%**

[1] Completeness was measured based on 107 marker genes.

[2] Contamination ratios were estimated using CheckM.

[3] The 0% completeness was due to the lack of marker genes that can be identified from this genome.

Supplementary Table 8. Primers used for GH gene cluster analysis and gene amplification

#	Position	Orientation	Sequence
01	5029F	Forward	ACTGGTACACGAAGAACTC
02	5929R	Reverse	GGGGTTTGCTGTTCAAAAG
03	8188F	Forward	TTCACGTATCACCGTTTCTG
04	8919R	Reverse	TTTTTTGATCCACTCTCCG
05	10392F	Forward	GTTCGATGAGCAATTAAAGG
06	11773R	Reverse	CAACCCACAAAACCTTCAT
07	14162F	Forward	GGCAGATCAGTTGGGTGAA
08	15135R	Reverse	GATCATGTTCAAGTCTCCG
09	15540F	Forward	AGACCAGTGTGTGGAATTTG
10	17489R	Reverse	AGACCAGTGTGTGGAATTTG
11	18031F	Forward	GCTGTGGGGCTATATTGAAG
12	19508R	Reverse	CGATACGGTGGTAGGCTT
13	2676F	Forward	CATATGCTGAGACGACGGGC
14	5892R	Reverse	CCTAgGGTTCGACGCCCCAG
15	6038F	Forward	CCATGGAATCACTCGCATGGA
16	8782R	Reverse	AAGCTTACGGTTCGACGCCCCA
17	8882F	Forward	CCATGGGAACGGGCCGTG
18	11631R	Reverse	AGATCTTTACGGCTCGACGCCCCAG
19	11830F	Forward	ATGACGATGGCGTGGAAACA
20	14844R	Reverse	AGATCTTTACGGCTCGACGCCCCAG
21	14947F	Forward	ATGAATCGACGCCTTATCGC
22	16902R	Reverse	TTATGGTTCAACGCCCCAGA
23	17189F	Forward	ATGATAATGCGAAACTGGCT
24	19213R	Reverse	TCAGGGTTCGACGCCCCAGAT

Supplementary Table 9. Exclusive unique spectrum counts are found for excised gel slices depicted in Supplementary Figure 8A-C. Exclusive unique spectrum counts are derived from excised gel slices from 2D-BN-PAGE samples from: anion-exchange chromatography **(A)**, affinity-digested supernatants denatured by heating **(B)** and without heat denaturation **(C)**. These unique spectrum counts are mapped on to sequences of CelABC and XynA in Supplementary Figure 9A-D and data for each peptide is provided in Supplementary Tables 10A-D.

(A)

	Exclusive unique spectrum count					
AIEX 2D C	CelA	CelB	CelC	XynA	S-Layer	
1	0	0	0	0	78	S-Layer
2	0	0	3	0	56	
3	0	2	34	0	5	CelC
4	28	29	2	0	0	CelA
5	1	36	0	0	0	CelB
6	0	5	7	20	4	XynA

(B)

	Exclusive unique spectrum count					
AD 2D C	CelA	CelB	CelC	XynA	S-Layer	
1	0	2	8	0	0	
2	15	17	31	0	0	
3	1	4	15	0	0	CelC
4	28	5	1	0	0	CelA
5	7	12	4	0	0	
6	2	60	2	0	0	CelB

(C)

	Exclusive unique spectrum count					
AD 2D NC	CelA	CelB	CelC	XynA	S-Layer	
1	30	2	38	0	0	C I
2	9	36	11	0	0	C II
3	0	30	1	0	0	C III
4	0	3	18	0	0	
5	3	9	4	2	0	

Abundant

Absent

Supplementary Table 10A. Sequences and properties of individual unique spectra of CelA. Mapping of these spectra to the CelA sequence is found in Supplementary Figure 9A.

Nr	Sequence	Prob	Mascot Ion score	Mascot Identity score	Mascot Delta Ion Score	Observed	Actual Mass	Charge	Delta Da	Delta PPM
1	(K)AIYFYDAQR(S)	97%	27.13	31.690865	25.52	573.8027	1,145.59	2	0.04035	35.19
2	(R)GDSGLNDGADVGVDLTGGWYDAGDHVK(F)	100%	32.56	29.20645	31.48	897.4243	2,689.25	3	0.06135	22.8
3	(R)DAFVQTGQLDYILNNIK(W)	100%	77.45	29.960737	70.96	976.5314	1,951.05	2	0.04282	21.94
4	(K)WATDYFIK(A)	99%	37.21	32.1801	20.97	522.2841	1,042.55	2	0.04122	39.5
5	(K)AHSAPNVLWGQVGK(G)	99%	22.81	28.095598	8.87	488.6169	1,462.83	3	0.06056	41.37
6	(R)GKYSDcITDAQNFYR(S)	100%	34.28	30.777311	34.28	613.2995	1,836.88	3	0.06644	36.15
7	(K)YSDcITDAQNFYR(S)	100%	57.41	28.350561	51.56	826.8743	1,651.73	2	0.04037	24.43
8	(K)AIASVAEWGR(E)	100%	64.13	30.909632	46.88	530.3028	1,058.59	2	0.04011	37.85
9	(R)EGQTPYWGYK(W)	95%	23.38	30.277573	16.18	614.806	1,227.60	2	0.04122	33.55
10	(K)WTQSWDDVHYGAQLLLAR(I)	100%	31.5	30.484419	31.5	720.3815	2,158.12	3	0.0629	29.13
11	(R)NLEYWTDGTDDTGER(I)	100%	64.23	27.589119	56.21	886.395	1,770.78	2	0.0418	23.59
12	(R)ITYTPGGLAWLDSWGSLR(Y)	100%	31.26	30.42969	31.26	665.0278	1,992.06	3	0.05081	25.49
13	(R)QVLYALGDNPR(N)	100%	48.07	30.538464	41.61	623.3528	1,244.69	2	0.03954	31.74
14	(R)NSSYVVGFGR(N)	100%	66.26	31.717264	58.96	543.2924	1,084.57	2	0.04009	36.93
15	(R)TAHGSWADSQNVPAYHR(H)	99%	26.8	30.799046	15.05	632.9838	1,895.93	3	0.06329	33.36
16	(R)HILYGALVGGPNQSDAYTDSISDYVGNEVATDYNAAFTGNLAK(M)	100%	94.25	26.36488	94.25	1,123.81	4,491.20	4	0.08069	17.96
17	(R)EDEFFVEAGVNSSGPNYTEIK(A)	100%	47.55	30.511524	47.55	778.0471	2,331.12	3	0.06516	27.94
18	(K)YFVDLSEVYAAGYTVNDIK(V)	100%	100.64	30.413929	99.96	1,084.05	2,166.09	2	0.04009	18.5
19	(K)VTTNYNEGAK(V)	100%	36.67	30.216026	27.1	548.7878	1,095.56	2	0.04146	37.81
20	(K)VSGLLPYDESRR(L)	100%	33.5	28.790958	30.45	464.6021	1,390.78	3	0.0638	45.84
21	(R)RLYYVLVDFTGTK(I)	100%	39.54	26.910816	36.24	525.6448	1,573.91	3	0.06202	39.38
22	(R)LSAPSGTSFWNPNNDFSYQLmSGTSNSSLVK(T)	100%	65.78	28.615343	57.67	1,118.21	3,351.60	3	0.06022	17.96
23	(K)TPYmPVYDAGVK(I)	100%	36.67	31.332195	26.76	678.8503	1,355.69	2	0.04285	31.58

Supplementary Table 10B. Sequences and properties of individual unique spectra of CelB. Mapping of these spectra to the CelB sequence is found in Supplementary Figure 9B.

Nr	Sequence	Prob	Mascot Ion score	Mascot Identity score	Mascot Delta Ion Score	Observed	Actual Mass	Charge	Delta Da	Delta PPM
1	(R)FLQLYNQIK(N)	100%	57.47	25	44.54	583.8575	1,165.70	2	0.05087	43.6
2	(K)mEQFIIPNSTEQPTmGSYNPSSPATYAPEHPYPDR(Y)	95%	31.02	27.176704	24.76	996.9761	3,983.88	4	0.1131	28.38
3	(R)YPTLLNNSVPAGQDPLDAELK(A)	100%	33.07	28.898619	28.27	752.4171	2,254.23	3	0.08114	35.98
4	(R)FGKPNEGFVTLFVK(D)	100%	50.01	25.976952	50.01	528.3181	1,581.93	3	0.07666	48.43
5	(K)DnGTPAQQWR(Y)	100%	42.57	31.325798	16.88	587.2929	1,172.57	2	0.05005	42.64
6	(R)YTAASDADAR(A)	100%	51.54	28.779469	37.39	520.7598	1,039.51	2	0.04797	46.1
7	(R)AIQVmYWAK(Q)	100%	48.9	29.180302	32.49	563.3163	1,124.62	2	0.04925	43.76
8	(K)QLGYnNQTYLDK(A)	100%	61.45	31.45507	13.7	729.3768	1,456.74	2	0.05552	38.09
9	(K)YFQQIGSANDGSPSPGSGK(N)	100%	55.33	30.937717	52.82	632.9872	1,895.94	3	0.07464	39.35
10	(R)IGSSHAHQGYQNPVAAYALSAGGLAPR(S)	100%	66.3	29.25312	65.19	898.4846	2,692.43	3	0.08478	31.48
11	(R)SATAQTDWATSLQR(Q)	100%	106.41	31.132746	95.54	768.4038	1,534.79	2	0.05546	36.11
12	(R)STFYGmVYDEAPVYR(D)	100%	47.65	30.930712	47.65	605.2996	1,812.88	3	0.07397	40.78
13	(R)VAELYYIWTSSGNTNTQQFQmVK(N)	100%	61.53	30.051805	61.53	908.7997	2,723.38	3	0.08694	31.91
14	(K)DPGQDVGVTGNYIK(L)	100%	60.12	31.486027	51.63	731.8889	1,461.76	2	0.05301	36.24
15	(K)LLTFFAAATK(A)	100%	55.43	25	47.2	541.8409	1,081.67	2	0.05002	46.2
16	(K)EIYFPSGWSGTYGQGNTIPGPGAVPSDPSK(G)	98%	28.41	29.556877	28.41	1,022.85	3,065.53	3	0.0852	27.78
17	(K)GGnGVYISYAELRPK(I)	100%	34.53	29.100904	32.32	542.3073	1,623.90	3	0.07414	45.63
18	(K)GGnGVYISYAELRPK(I)	100%	32.54	29.100904	32.48	542.3073	1,623.90	3	0.07414	45.63
19	(K)WSYLENLYK(T)	99%	39.72	32.10051	30.65	608.3313	1,214.65	2	0.05094	41.9
20	(R)WENGVPTFTYHR(F)	100%	25.88	31.290451	25.88	502.9334	1,505.78	3	0.07333	48.66
21	(R)FWAQVDVATAYAEFAR(L)	100%	98.69	31.2156	88.68	922.981	1,843.95	2	0.05821	31.55

Supplementary Table 10C. Sequences and properties of individual unique spectra of CelC. Mapping of these spectra to the CelC sequence is found in Supplementary Figure 9C.

Nr	Sequence	Prob	Mascot Ion score	Mascot Identity score	Mascot Delta Ion Score	Observed	Actual Mass	Charge	Delta Da	Delta PPM
1	(R)VSDPTLAAK(M)	100%	41.66	31.479853	14.22	451.2747	900.5349	2	0.04312	47.82
2	(K)TYPTAVWLDR(I)	96%	26.53	29.973866	18.12	611.3416	1,220.67	2	0.04954	40.55
3	(R)IAAIDGGPGR(R)	99%	31.3	27.979595	11.39	463.7792	925.5438	2	0.04543	49.03
4	(R)SLVQHLDTALAQK(Q)	100%	42.5	25.622929	39.24	475.2919	1,422.85	3	0.0707	49.65
5	(K)QGNTPITAmFVIYNmPGR(D)	100%	30.72	30.65953	30.72	681.3586	2,041.05	3	0.07753	37.96
6	(R)DcAALASnGELPLTQEGLQR(Y)	100%	82.62	30.989897	27.15	1,072.55	2,143.08	2	0.06198	28.91
7	(R)YKTEYIDR(I)	99%	34.3	32.135178	22.4	544.2984	1,086.58	2	0.04752	43.69
8	(R)IAAIFADPK(Y)	100%	65.63	26.28389	56.01	473.297	944.5794	2	0.04614	48.8
9	(R)IVTVIEPDGLPNLVTNLSDPEcAQANSSGIYVEAVR(Y)	100%	43.42	27.041504	43.42	1,281.01	3,840.01	3	0.08936	23.26
10	(R)TGAVQLYTNVVR(G)	100%	61.13	27.015678	46.14	660.893	1,319.77	2	0.05163	39.09
11	(K)GLSSVDGFVTNVANYTPLEEPYLTDPNLTVGGQPLK(S)	100%	45.09	27.024305	45.09	1,269.34	3,805.00	3	0.09073	23.84
12	(K)FYEWNPYFDEVDYAAALR(S)	100%	73.75	30.666985	72.1	757.0409	2,268.10	3	0.08448	37.23
13	(R)SAFISAGWPTSIGmLIDTSR(N)	100%	70.35	29.986952	70.35	709.3843	2,125.13	3	0.0796	37.44
14	(R)nGWGGPNRPTGASGTTVDAYVNSGR(V)	100%	37.62	30.622059	7.56	831.4173	2,491.23	3	0.08237	33.05
15	(R)VVGNQLVDQnGQPIQLR(G)	100%	63.07	26.560982	10.85	627.0332	1,878.08	3	0.08151	43.37
16	(R)GISSHGLQWYGHFVNR(D)	97%	20.14	30.77368	20.14	620.0024	1,856.99	3	0.07824	42.11
17	(R)WLRDDWGITVFR(A)	98%	19.13	28.847954	19.13	521.9648	1,562.87	3	0.07286	46.59
18	(R)DDWGITVFR(A)	100%	52.96	30.965624	30.2	554.7987	1,107.58	2	0.04779	43.11
19	(R)AALYTAEQGYITNPSLK(E)	100%	74.76	29.80912	74.76	920.5053	1,839.00	2	0.0545	29.62
20	(K)AFFDEmSR(L)	99%	36.98	30.182842	28.08	509.7421	1,017.47	2	0.04712	46.26
21	(R)QISWVNWSLSDK(A)	98%	35.7	31.367207	27.18	731.8924	1,461.77	2	0.045	30.76
22	(K)AESSAALLPGASATGGWTDAQLSQSGR(F)	100%	90.27	30.051805	88.07	863.7831	2,588.33	3	0.08055	31.11

Supplementary Table 10D. Sequences and properties of individual unique spectra of XynA. Mapping of these spectra to the XynA sequence is found in Supplementary Figure 9D.

Nr	Sequence	Prob	Mascot Ion score	Mascot Identity score	Mascot Delta Ion Score	Observed	Actual Mass	Charge	Delta Da	Delta PPM
1	(R)GGVETLTVTSAAAYSGAYGLSVSGR(T)	100%	84.71	30.591846	84.71	792.0534	2,373.14	3	-0.04276	-18.01
2	(K)TYQFSAWVK(L)	100%	42.17	30.305998	40.07	565.2792	1,128.54	2	-0.01651	-14.62
3	(K)LPSGSSNTR(I)	99%	32.92	31.62863	20.78	459.7279	917.4413	2	-0.01533	-16.69
4	(R)IEQLPDLPK(T)	100%	41.22	29.074114	21.47	526.795	1,051.58	2	-0.01613	-15.33
5	(K)TVEENIPSLK(D)	100%	58.88	32.06556	28.64	565.3008	1,128.59	2	-0.01564	-13.84
6	(R)FPIGTAFENFELLDEQDR(K)	100%	86.66	28.847954	82.31	1,070.99	2,139.98	2	-0.03638	-16.99
7	(K)HFNSVTPGNVLK(W)	98%	28.21	31.024338	21.65	656.8444	1,311.67	2	-0.01934	-14.73
8	(K)AVAFAVQnGmK(I)	100%	56.5	30.182842	13.75	576.7813	1,151.55	2	-0.01655	-14.36
9	(K)GIIYAWDVVNEVIDPGQPDGLR(R)	100%	72.54	30.614525	71.93	1,213.60	2,425.18	2	-0.04448	-18.33
10	(R)SLWYQIAGEEYIEK(A)	100%	63.61	29.88113	60.33	864.9132	1,727.81	2	-0.02884	-16.68
11	(K)AFQFAHEADPNALLFINDYNTHESGK(S)	100%	46.96	27.788744	41.49	983.7835	2,948.33	3	-0.04437	-15.05
12	(K)SQALYNLVQR(L)	100%	52.9	31.829851	27.08	596.319	1,190.62	2	-0.01726	-14.48
13	(K)FSNLGVVQEITELDmSIYNNSSQK(Y)	100%	95.19	29.13284	93.23	911.429	2,731.27	3	-0.03615	-13.23
14	(K)YDTLPSDLAQQQATR(Y)	100%	65.53	30.090258	58.97	853.9085	1,705.80	2	-0.02491	-14.59
15	(R)QLFEmFLR(R)	99%	34.37	29.484129	23.92	550.2752	1,098.54	2	-0.01742	-15.85
16	(R)RSSLIQNVTFWGK(D)	95%	28.89	31.1227	20.8	512.6057	1,534.80	3	-0.03036	-19.77
17	(K)DDANTWLR(K)	99%	53.03	29.14343	38.16	495.7286	989.4426	2	-0.01417	-14.3
18	(R)NDWPLLFDEQLK(A)	100%	63.96	31.0721	57.62	759.3746	1,516.73	2	-0.02182	-14.37

Supplementary Table 11. GC-MS quantification of trimethylsilyl-derivatives of sugars from preparation from affinity digestion (AD). This analysis was performed once on a single sample.

Glycosyl residue	Mass (µg)	Mol %
Ribose (Rib)	0.0	0.9
Xylose (Xyl)	0.0	0.5
Mannose (Man)	0.2	3.2
Galactose (Gal)	4.4	72.3
Glucose (Glc)	0.6	9.7
N-acetyl galactosamine (GalNAc)	0.6	8.7
N-acetyl glucosamine (GlcNAc)	0.3	4.6

Supplementary Table 12. IMG IDs for metagenomic sequencing data.

Sample	Day	Sample name	IMG Genome ID
15L cultivation			
	1	B1F01	3300005156
	2	B1F02	3300005116
	3	B1F03	3300005117
	4	B1F04	3300005113
	5	B1F05	3300005108
	6	B1F06	3300005152
	7	B1F07	3300005153
	8	B1F08	3300005151
	9	B1F09	3300005150
	10	B1F10	3300005112
	11	B1F11	3300005155
	13	B1F13	3300005154
	14	B1F14	3300005135
	15	B1F15	3300005133
300 L cultivation			
	4	D2F03	3300005109
	5	D2F04	3300005149
	7	D2F06	3300005111
	10	D2F08	3300005107
	12	D2F10	3300005110
	14	D2F12	3300005136

<u>**Supplementary Figures**</u>

Supplementary Figure 1.

Cultivation of cellulolytic consortium at 300 L scale. (A) Daily relative abundances (calculated using average number of reads of binned scaffolds from time series metagenomic data of 300 L cultivation) of the *Paenibacillaceae* 1 population (red) during a 14 day cultivation of consortium grown with microcrystalline cellulose. **B)** CMCase (red) and xylanase (green) activity measurements obtained by daily sampling of 300 L cultivation. Enzymatic assays are reported as the mean of technical replicates (n=3) and error bars represent the standard error of the mean. **(C)** Relative abundance of dominant populations (≥1%) at day 14.

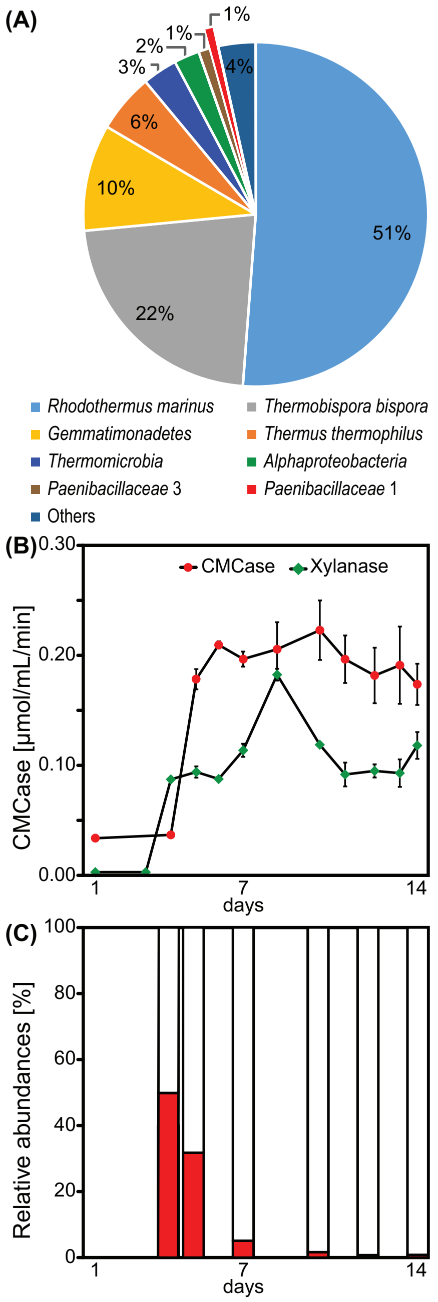

Supplementary Figure 2

Ribosomal RNA maximum-likelihood trees. Maximum-likelihood phylogenetic trees based on **(A)** 16S and **(B)** 23S rRNA gene sequences showing the phylogenetic affiliation of '*Candidatus* Reconcilibacillus cellulovorans' and other selected references. *Clostiridum botulinium* and *Clostridium perfringens* served as outgroups.

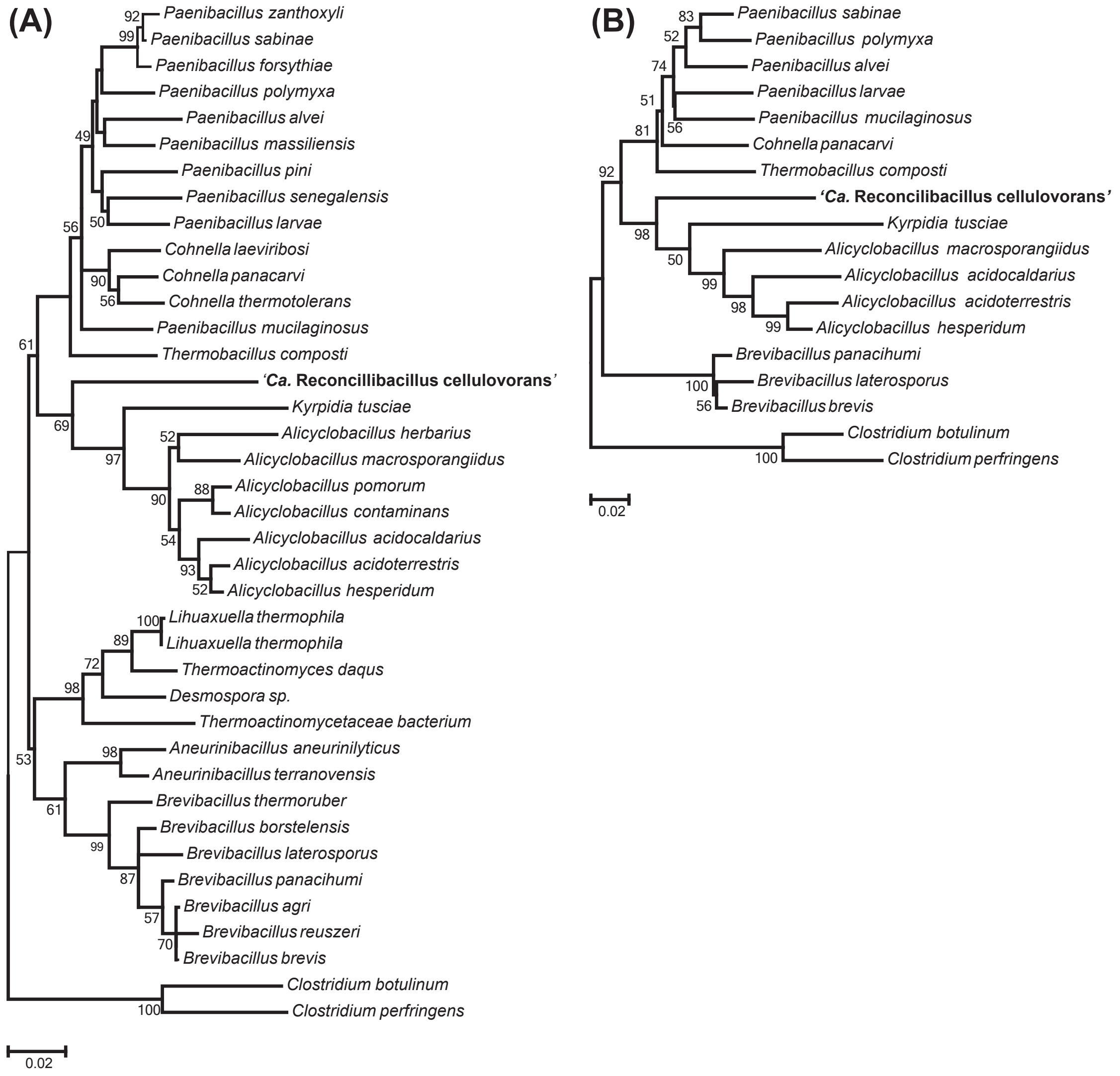

Supplementary Figure 3

DNA polymerase (PolA) maximum-likelihood trees. Maximum-likelihood phylogenetic tree is based on the amino acid sequence of PolA. *Geobacter uraniireducens* and *Geobacter bemidjiensis* served as outgroups.

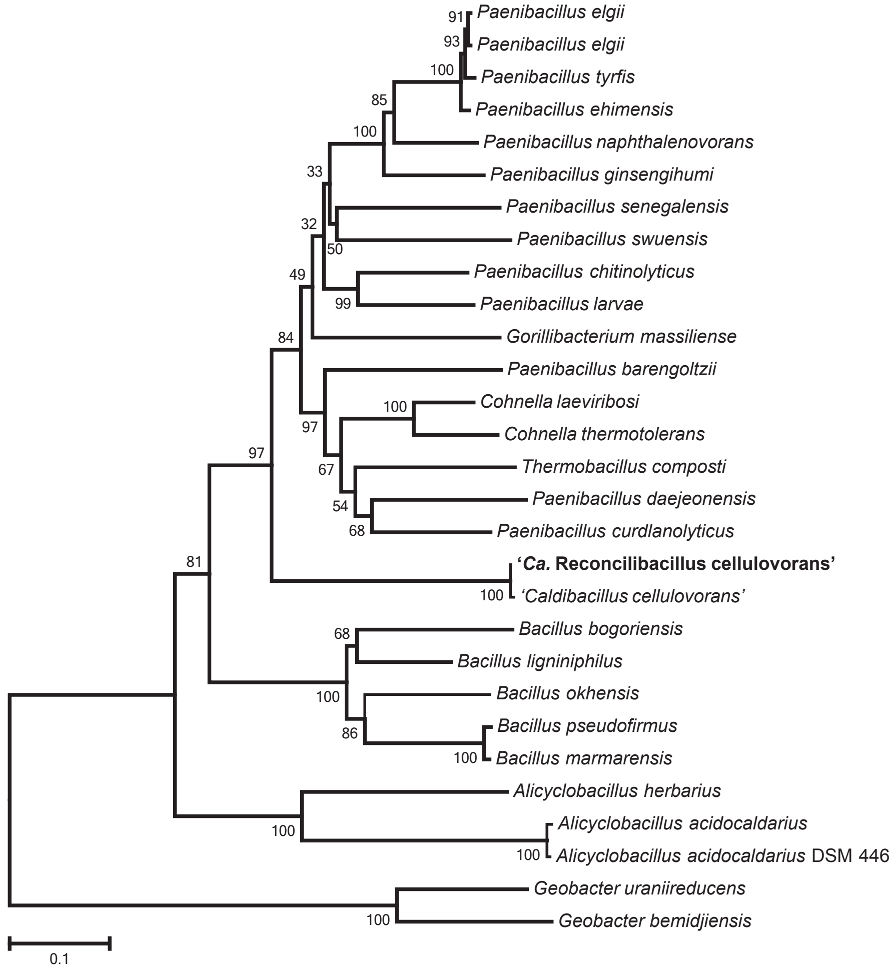

Supplementary Figure 4

Reconstruction of '*Ca.* Reconcilibacillus cellulovorans' gene cluster. Schematic organization of the glycoside hydrolase gene cluster of '*Ca.* Reconcilibacillus cellulovorans' (orange) with its CDS (yellow). Schematic map of metagenomic contigs (red) connected through PCR fragments (green) and confirmed by amplicons of individual glycoside hydrolases (blue). Primer binding positions and orientations are indicated by arrows. Primer sequences are provided in Supplementary Table 8.

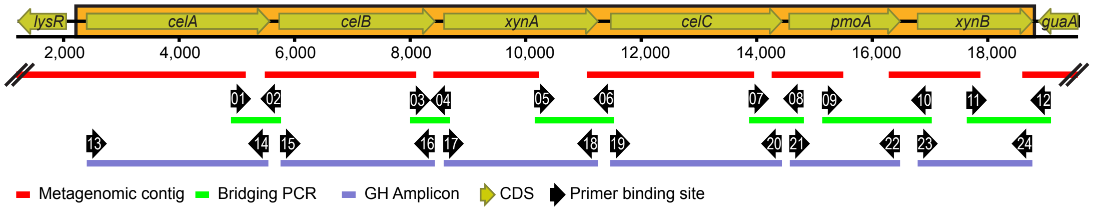

Supplementary Figure 5

Comparison of glycoside hydrolase gene clusters. Molecular organization of glycoside hydrolase gene clusters of '*Ca.* Reconcilibacillus cellulovorans', *Caldicellulosiruptor bescii*, *Caldicellulosiruptor obsidiansis*, and *Caldicellulosiruptor saccharolyticus*. CDS coding for glycoside hydrolases (yellow) are complemented with individual active sites (grey), poly-proline stretches (green), and cellulose binding motifs (red). CDS encoding non-GHs are indicated in brown. Genomic data for *Caldicellulosiruptor* isolates was obtained from IMG (https://img.jgi.doe.gov/).

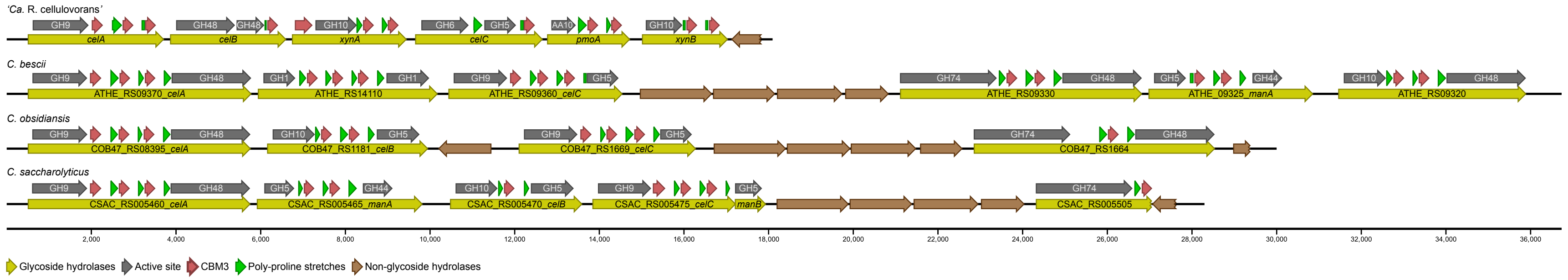

Supplementary Figure 6

Maximum-likelihood phylogenetic trees for catalytic domains. Maximum-likelihood phylogenetic trees based on amino-acid sequences of catalytic domains of glycoside hydrolases of ’*Ca.* Reconcilibacillus cellulovorans’ including (A) GH9, (B) GH48, (C) GH10, (D) GH6, (E) GH5, and (F) AA10.

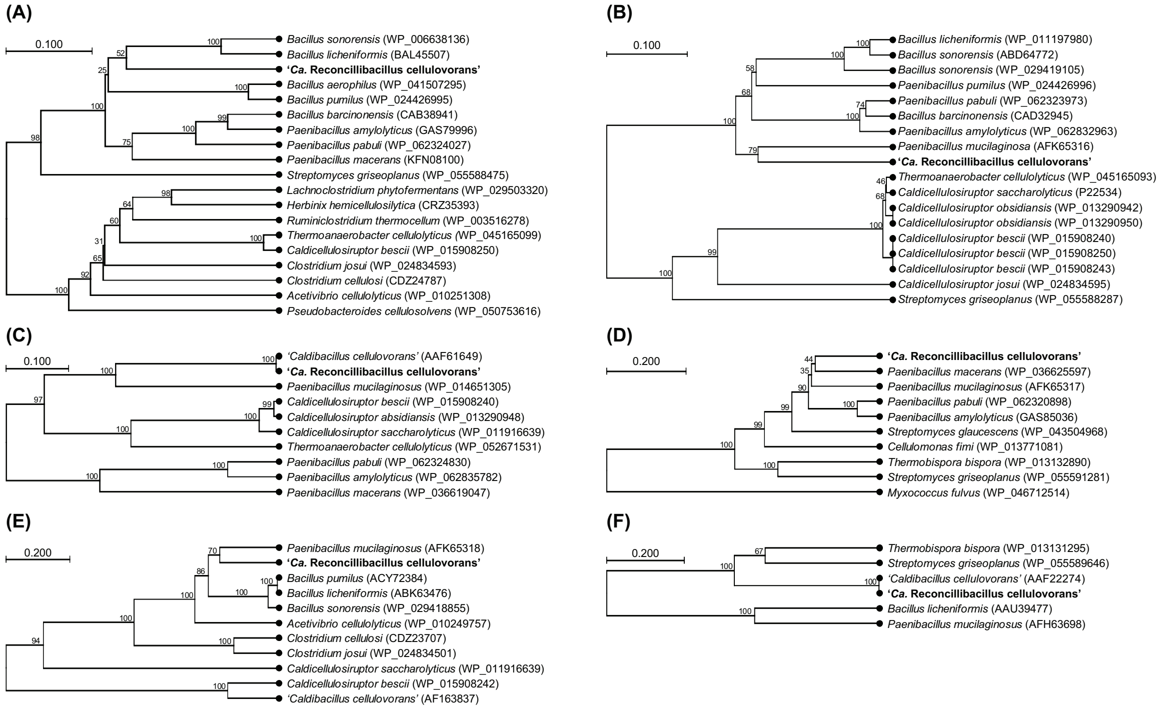

Supplementary Figure 7

Chromatographic separation of glycoside hydrolase activties. Protein concentrations (solid) and enzymatic activities (lines) with CMC, xylan, pNP-glucose, pNP-cellobiose, pNP-xylose, and pNP-arabinose substrates were measured for each fraction of the **(A)** supernatant and **(B)** affinity-digested supernatant separated by anion-exchange chromatography. Enzymatic assays are reported as the mean of technical replicates (n=3) and error bars represent the standard error of the mean. These traces are representative of separations by anion exchange chromatography that were performed five times.

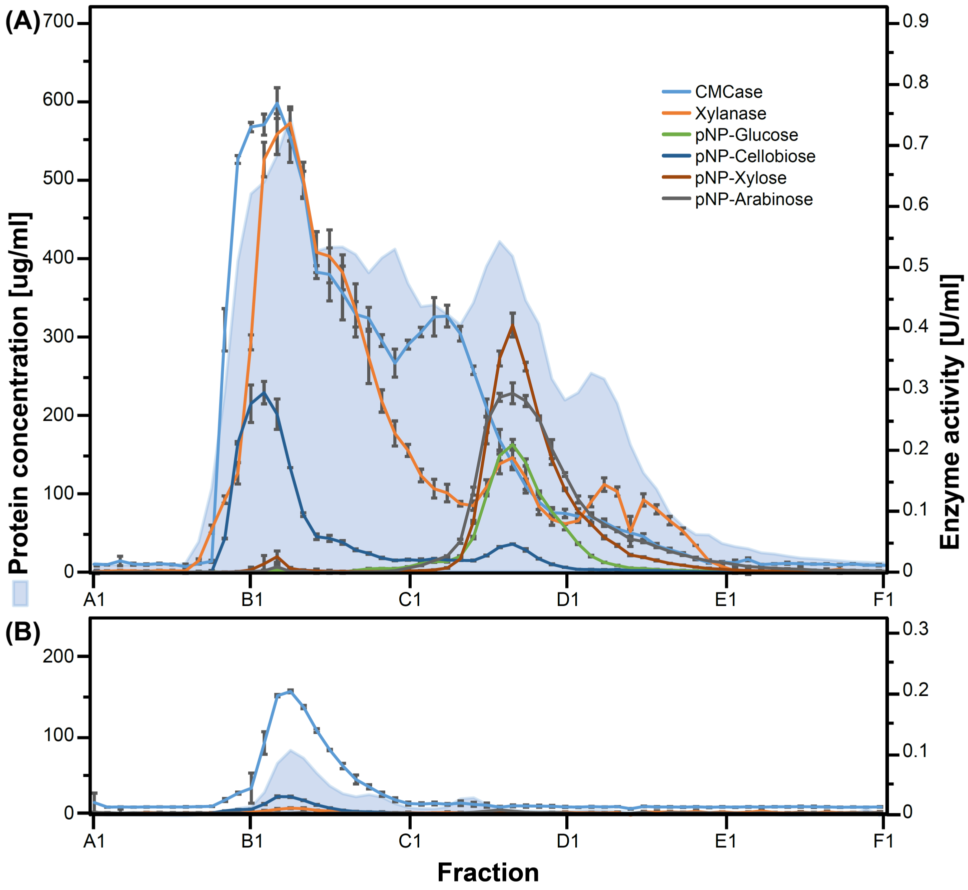

Supplementary Figure 8

Proteomic analysis. Proteomic analysis of excised gel slices from 2D-BN-PAGE samples from: anion-exchange chromatography **(A)**, affinity-digested supernatants denatured by heating **(B)** and without heat denaturation **(C)**. These images are identical to the gel images in Figure 3. Excised gel slices are indicated by squares and their exclusive unique spectrum counts are found in Supplementary Table 9. These proteomics measurements are representative of three sets of measurements performed on the protein fractions separated by 2-D BN-PAGE and SDS-PAGE.

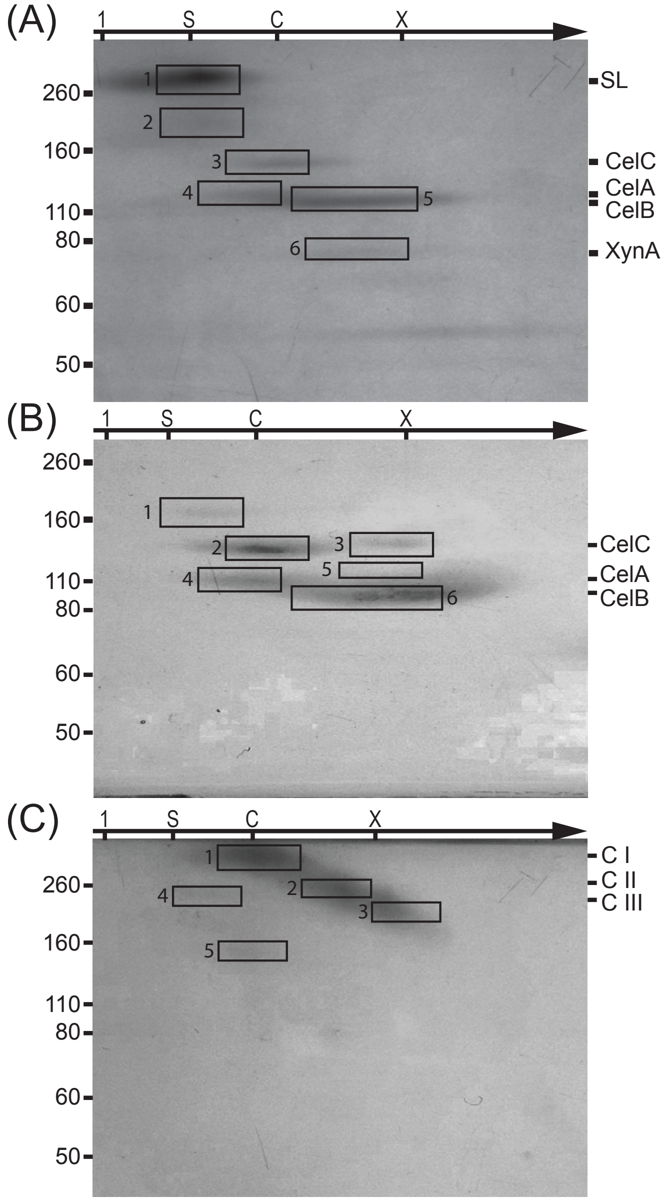

Supplementary Figure 9A

CelA Proteomics. CelA amino-acid sequence with exclusive unique spectra are indicated by orange and yellow; the yellow distinguishes adjacent detected peptides. Data supporting the assignment of these peptides is found in Supplementary Table 9A.

```
CelA  MLRRRALSMLTGAAVVYSAFVPVGSPDSAVVRAAPTSYNYAEALQKAIYFYDAQRSGKLPPDNRVEWRGDSGLNDGADVGVDLTGGWYDAGDHVKFGLPM
CelA  AYSAAMLAWAVYEYRDAFVQTGQLDYILNNIKWATDYFIKAHSAPNVLWGQVGKGDVDHAWWGPAEVMQMPRPAYKIDPSCPGSDLAAGTAAAMAAAAAV
CelA  FKPTDPTYASTLIAHAKQLYTFADTYRGKYSDCITDAQNFYRSWSGYADELTWGAVWLYLATGEQAYLDKAIASVAEWGREGQTPYWGYKWTQSWDDVHY
CelA  GAQLLLARITGDQRFIQSTERNLEYWTDGTDDTGERITYTPGGLAWLDSWGSLRYAMNASFLAFVYSDWLQSRDPAKAEKYRNFAVRQVLYALGDNPRNS
CelA  SYVVGFGRNPPQRPHHRTAHGSWADSQNVPAYHRHILYGALVGGPNQSDAYTDSISDYVGNEVATDYNAAFTGNLAKMYLLFGASAGQRPLANFPEPEVR
CelA  EDEFFVEAGVNSSGPNYTEIKALINNRSGWPARMGDKLSFKYFVDLSEVYAAGYTVNDIKVTTNYNEGAKVSGLLPYDESRRLYYVLVDFTGTKIYPGGQ
CelA  SAYKKEVQFRLSAPSGTSFWNPNNDFSYQLMSGTSNSSLVKTPYMPVYDAGVKIFGVEPSSGSGSSPTPPPTSTPTPTPTPTPTPTLTLTPTPTPTPTPT
CelA  PTPTPTPTPTPTPTPTPTPTPTPTPTPTPTPTPTPTPTPSASGTLRVEYRVGDTSATDNQMKPYLRIVNTGSQAVPLTELKVRYWYTKNSTQAEQYFCDW
CelA  AQIDCSNIRAQFVSLAQPVSGADSYIELSFTGGSVPAGGNTGEIQNRIHFTNWMNYNETDDWSYNGTQTTWGPSTRITLYRNGVLWWGTEPGGGSSTPTP
CelA  TATPTPTPSAAPTPTPTAGGSLVVQYRAADTNAGDNQLKPHFRIVNRGTTSVPLSELSIRYWYTVDGDKPQVFNCDWAQVGCSNLRGSFVKLSTGRTGAD
CelA  YYIEITFTSGAGSLAAGGSSGDIQVRINKNDWTNYNEANDYSYDPTKTSFADWNRVTLYRNGQLIWGVEP*
```

Supplementary Figure 9B

CelB Proteomics. CelB amino-acid sequence with exclusive unique spectra are indicated by orange and yellow; the yellow distinguishes adjacent detected peptides. Data supporting the assignment of these peptides is found in Supplementary Table 9B.

```
CelB MESLAWTLLWKKARIIFLAFALVVSAFAGFAVSPRSETAYAQTDPQVFKDRFLQLYNQIKNPANGYFSPEGIPYHSIETLISEAPDYGHMTTSEAFSYWL
CelB WLETLYGYFTGDWSKLEQAWTKMEQFIIPNSTEQPTMGSYNPSSPATYAPEHPYPDRYPTLLNNSVPAGQDPLDAELKATYGNNVTYLMHWLLDVDNWYG
CelB FGNLLNPSHTATYVNTFQRGEQESVWEAITHPSQDNFRFGKPNEGFVTLFVKDNGTPAQQWRYTAASDADARAIQVMYWAKQLGYNNQTYLDKARKMGDY
CelB LRYTLFDKYFQQIGSANDGSPSPGSGKNSAHYLLSWYTAWGGGLGSGGNWAWRIGSSHAHQGYQNPVAAYALSAGGLAPRSATAQTDWATSLQRQLEFYT
CelB WLQSSEGAIGGGATNSVGGSYQPYPSGRSTFYGMVYDEAPVYRDPPSNSWFGFQAWSVERVAELYYIWTSSGNTNTQQFQMVKNIVTKWVDWALDYTFVN
CelB QRPVTDAQGYFLTSSGSRVLGGNNPQIATVSDPGQFYIPSTLEWQGQPDTWNGYANYTGNPNFHAIAKDPGQDVGVTGNYIKLLTFFAAATKAETGNYTA
CelB LGSQALNVAEQLLNVLWNFNDGVGIVRPEQRADYFRYFTKEIYFPSGWSGTYGQGNTIPGPGAVPSDPSKGGNGVYISYAELRPKIKQDPKWSYLENLYK
CelB TSYNPSTGRWENGVPTFTYHRFWAQVDVATAYAEFARLIGGLGASPTPTPSATPTPTPSAGGNLVVQYRAADTNATDNQLKPHFRIVNRGTTSVPLSELT
CelB IRYWYTVDGDKPQVFNCDWAQVGCSNVRGSFVKLTTGRTGADYYIEITFTSGAGSLAAGGSSGDIQVRINKNDWTNYNEANDYSCDPTKTSFADWNRVTL
CelB YRNGQLVWGVEP*
```

Supplementary Figure 9C

CelC Proteomics. CelC amino-acid sequence with exclusive unique spectra are indicated by orange and yellow; the yellow distinguishes adjacent detected peptides. Data supporting the assignment of these peptides is found in Supplementary Table 9C.

```
                         20                  40                  60                  80                 100
CelC MTMAWKQRSGLIALILALVAGLLLPWGSLPKAAAEPHVDNPFVGATAYVNPDYAALVDSSIARVSDPTLAAKMRTVKTYPTAVWLDRIAAIDGGPGRRSL
                        120                 140                 160                 180                 200
CelC VQHLDTALAQKQGNTPITAMFVIYNMPGRDCAALASNGELPLTQEGLQRYKTEYIDRIAAIFADPKYAGIRIVTVIEPDGLPNLVTNLSDPECAQANSSG
                        220                 240                 260                 280                 300
CelC IYVEAVRYAINKLSEIPNVYIYLDIAHSGWLGWDNNRTGAVQLYTNVVRGTTKGLSSVDGFVTNVANYTPLEEPYLTDPNLTVGGQPLKSAKFYEWNPYF
                        320                 340                 360                 380                 400
CelC DEVDYAAALRSAFISAGWPTSIGMLIDTSRNGWGGPNRPTGASGTTVDAYVNSGRVDRRAHRGLWCNVSGAGMGMPPQVAPAAYASQGIEAFVWVKPPGE
                        420                 440                 460                 480                 500
CelC SDGASSEIPNDEGKRFDRMCDPTYTTQYGVLTGALPNAPLAGQWFHDQFVMLVQNAYPAIPTSGGGTPTPSTTVTPTPTPTPTPTPSATVTPTPTPTPTP
                        520                 540                 560                 580                 600
CelC TPSATVTPTPTPTPTPTPTVTPTPTSSTSFVARHGQLRVVGNQLVDQNGQPIQLRGISSHGLQWYGHFVNRDSLRWLRDDWGITVFRAALYTAEQGYITN
                        620                 640                 660                 680                 700
CelC PSLKEKVKEAVQAAIELGIYVIIDWHILSDGDPNTYKEQAKAFFDEMSRLYGSYPNVIYEIANEPNGVTWEGQVKPYASEVIPVIRANDPDNLIIVGTTT
                        720                 740                 760                 780                 800
CelC WSQDVHLAADSPLPYSNLAYALHFYAGTHGQWLRDRIDYARNKGIAIFVSEWGTSTSTGDGGPYLTESQQWLDFLNARQISWVNWSLSDKAESSAALLPG
                        820                 840                 860                 880                 900
CelC ASATGGWTDAQLSQSGRFVRAQIRSGVLTPTPTPTPTPTPSAAPTPTPTAGGSLVVQYRAADTNAGDNQLKPHFRIVNRGTTSVPLSELSIRYWYTVDGD
                        920                 940                 960                 980               1,000
CelC KPQVFNCDWAQVGCSNLRGSFVKLSTGRTGADYYIEITFTSGAGSLAAGGSSGDIQVRINKNDWTNYNEANDYSYDPTKTSFADWNRVTLYRNGQLIWGV

CelC EP*
```

Supplementary Figure 9D

XynA Proteomics. XynA amino-acid sequence with exclusive unique spectra are indicated by orange and yellow; the yellow distinguishes adjacent detected peptides. Data supporting the assignment of these peptides is found in Supplementary Table 9D.

```
                    20                  40                  60                  80                 100
XynA MLLVLAIGLLLPIPYLHVASAENVLILQSDFEDGTTQGWVGRGGVETLTVTSAAAYSGAYGLSVSGRTKTWHGPTLDITSYIQVGKTYQFSAWVKLPSGS
                   120                 140                 160                 180                 200
XynA SNTRIYMTMQRTMQDTVYYEQIYFDTASAGNWVQLKAQYKLYEPAVNLQVYFEAPDHATQSFYIDDVRIEQLPDLPKTVEENIPSLKDVFAGRFPIGTAF
                   220                 240                 260                 280                 300
XynA ENFELLDEQDRKLILKHFNSVTPGNVLKWDSTEPQEGVFNFTESDKAVAFAVQNGMKIRGHTLIWHNQTPNWVFYDSNGNLVSKEVLYQRMERHIKPVVS
                   320                 340                 360                 380                 400
XynA RYKGIIYAWDVVNEVIDPGQPDGLRRSLWYQIAGEEYIEKAFQFAHEADPNALLFINDYNTHESGKSQALYNLVQRLKNKGIPVHGVGHQTHINISWPSI
                   420                 440                 460                 480                 500
XynA SEIENSLVKFSNLGVVQEITELDMSIYNNSSQKYDTLPSDLAQQQATRYRQLFEMFLRRSSLIQNVTFWGKDDANTWLRKFPVVRNDWPLLFDEQLKAKP
                   520                 540                 560                 580                 600
XynA AYWAVVGTVPSPTPTPTSTATPTPTPTVIPTPTPTPTPTSTPTPTPTSTPTPSASGTLRVEYRVGDSSATDNQMKPQLRIVNTGSQAVPLTELKVRYWYT
                   620                 640                 660                 680                 700
XynA KNSTQAEQYFCDWAQIGCSNIRAQFVSLAQPVSGADSYIELSFTGGSVPAGGNTGEIQNRIHFTNWMNYNETDDWSYNGTQTTWGPSTRITLYRNGVLVW
                   720                 740                 760                 780                 800
XynA GTEPGGGSPTPTPTPTSTPTPTSTPTPTPTPTPTPTATPTPTPTPTPSAGGNLVVQYRAADTNAGDNQLKPHFRIVNRGTTSVPLSELTIRYWYTVDGDK
                   820                 840                 860                 880                 900
XynA PQVFNCDWAWVGCSNLRGSLVKLTTGRTGADYYLEITFTSGAGSLAPGANSGDIQARINKNDWTNYNEANDYSYDPTKTSFADWNRVTLYRNGQLVWGVE

XynA P*
```

Supplementary Figure 10

Evidence for glycoside hydrolase glycosylation. Coomassie stain and glycoprotein stain of SDS-PAGE of CelABC complexes obtained by affinity digestion. Lane 1: MW markers (kDa); Lane 2: Coomassie staining of AD fraction (7.5 µg); Lane 3: periodic acid-Schiff base staining of AD fraction (7.5 µg). CelC and CelB are identified in Lane 3. The gel was run at 140 V for 3 h to separate CelA, which is not glycosylated, and CelB. This gel was representative of four gels representing four technical replicates of the glycosylation stain. The image is cropped for clarity and the original gel image is in Supplementary Figure 16.

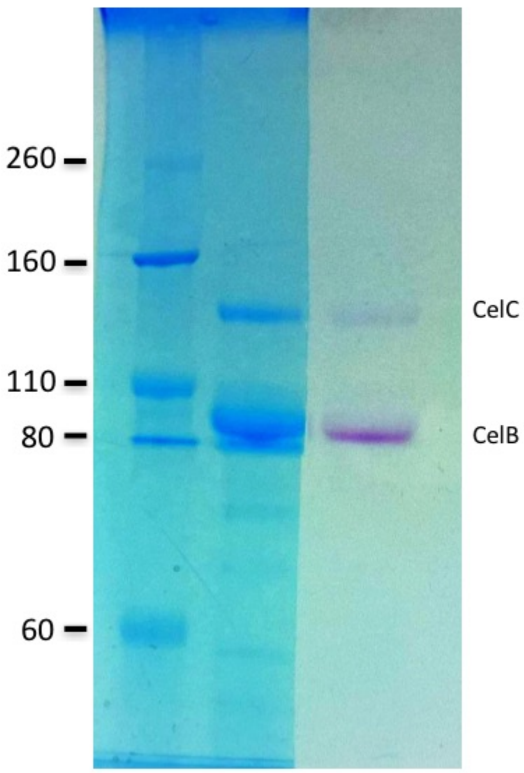

Supplementary Figure 11

Beta-elimination to identify O-linked glycans. A) Annotated MALDI-TOF MS spectrum of permethylated glycans from the sample obtaining via beta-elimination. All annotated ions are $[M+Na]^+$. The number indicated above the peak in the spectra is the m/z value of the ion that has been detected by the mass spectrometer. Because the linkage (linear or branched) of the galactoses in these glycans is not clear, the annotation is simplified by using a disaccharide with the remaining hexoses listed outside a bracket. **B)** Annotated ESI MS/MS spectrum of the permethylated glycan at m/z 493.2 from the sample obtaining via beta elimination. Assignments of the possible fragment ions are indicated on the cartoons and on the spectra the horizontal arrows show antennae losses. The number indicated above the peak is the m/z value of the fragment ion (resulting ion) that has been detected by the mass spectrometer. Data were acquired in forms of [M+Na]+. The fragment ions 241.1, 271.1, 259.1 and 275.2 exclusively provides evidence for the presence of two permethylated galactoses. The fragment ions yielded from permethylated xylose and heptose were not observed. The galactose monomers are depicted in yellow. This analysis was performed once on a single sample.

A)

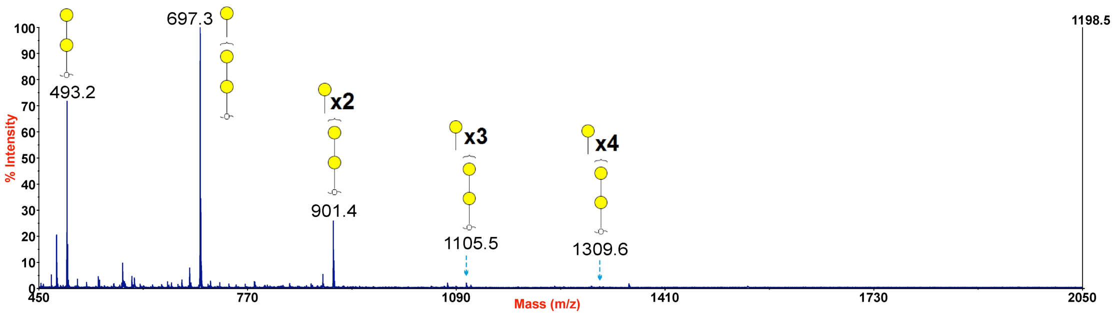

B)

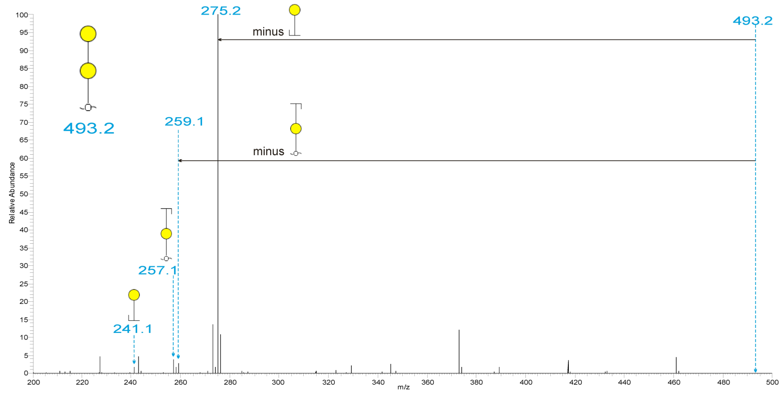

Supplementary Figure 12

Predicted glycosylation sites in CelB and CelC. Amino acids sequences of CelB and CelC with Ser-Pro-Thr linkers in bold. **A)** CelB; **B)** CelC.

A) CelB

MESLAWTLLWKKARIIFLAFALVVSAFAGFAVSPRSETAYAQTDPQVFKDRFLQLYNQIK
NPANGYFSPEGIPYHSIETLISEAPDYGHMTTSEAFSYWLWLETLYGYFTGDWSKLEQAW
TKMEQFIIPNSTEQPTMGSYNPSSPATYAPEHPYPDRYPTLLNNSVPAGQDPLDAELKAT
YGNNVTYLMHWLLDVDNWYGFGNLLNPSHTATYVNTFQRGEQESVWEAITHPSQDNFRFG
KPNEGFVTLFVKDNGTPAQQWRYTAASDADARAIQVMYWAKQLGYNNQTYLDKARKMGDY
LRYTLFDKYFQQIGSANDGSPSPGSGKNSAHYLLSWYTAWGGGLGSGGNWAWRIGSSHAH
QGYQNPVAAYALSAGGLAPRSATAQTDWATSLQRQLEFYTWLQSSEGAIGGGATNSVGGS
YQPYPSGRSTFYGMVYDEAPVYRDPPSNSWFGFQAWSVERVAELYYIWTSSGNTNTQQFQ
MVKNIVTKWVDWALDYTFVNQRPVTDAQGYFLTSSGSRVLGGNNPQIATVSDPGQFYIPS
TLEWQGQPDTWNGYANYTGNPNFHAIAKDPGQDVGVTGNYIKLLTFFAAATKAETGNYTA
LGSQALNVAEQLLNVLWNFNDGVGIVRPEQRADYFRYFTKEIYFPSGWSGTYGQGNTIPG
PGAVPSDPSKGGNGVYISYAELRPKIKQDPKWSYLENLYKTSYNPSTGRWENGVPTFTYH
RFWAQVDVATAYAEFARLIGGLGA**SPTPTPSATPTPTPS**AGGNLVVQYRAADTNATDNQL
KPHFRIVNRGTTSVPLSELTIRYWYTVDGDKPQVFNCDWAQVGCSNVRGSFVKLTTGRTG
ADYYIEITFTSGAGSLAAGGSSGDIQVRINKNDWTNYNEANDYSCDPTKTSFADWNRVTL
YRNGQLVWGVEP

B) CelC

MTMAWKQRSGLIALILALVAGLLLPWGSLPKAAAEPHVDNPFVGATAYVNPDYAALVDSS
IARVSDPTLAAKMRTVKTYPTAVWLDRIAAIDGGPGRRSLVQHLDTALAQKQGNTPITAM
FVIYNMPGRDCAALASNGELPLTQEGLQRYKTEYIDRIAAIFADPKYAGIRIVTVIEPDG
LPNLVTNLSDPECAQANSSGIYVEAVRYAINKLSEIPNVYIYLDIAHSGWLGWDNNRTGA
VQLYTNVVRGTTKGLSSVDGFVTNVANYTPLEEPYLTDPNLTVGGQPLKSAKFYEWNPYF
DEVDYAAALRSAFISAGWPTSIGMLIDTSRNGWGGPNRPTGASGTTVDAYVNSGRVDRRA
HRGLWCNVSGAGMGMPPQVAPAAYASQGIEAFVWVKPPGESDGASSEIPNDEGKRFDRMC
DPTYTTQYGVLTGALPNAPLAGQWFHDQFVMLVQNAYPAIPTSGGG**TPTPSTTVTPTPTP**
TPTPTPSATVTPTPTPTPTPTPSATVTPTPTPTPTPTPTVTPTPTSSTSFVARHGQLRVV
GNQLVDQNGQPIQLRGISSHGLQWYGHFVNRDSLRWLRDDWGITVFRAALYTAEQGYITN
PSLKEKVKEAVQAAIELGIYVIIDWHILSDGDPNTYKEQAKAFFDEMSRLYGSYPNVIYE
IANEPNGVTWEGQVKPYASEVIPVIRANDPDNLIIVGTTTWSQDVHLAADSPLPYSNLAY
ALHFYAGTHGQWLRDRIDYARNKGIAIFVSEWGTSTSTGDGGPYLTESQQWLDFLNARQI
SWVNWSLSDKAESSAALLPGASATGGWTDAQLSQSGRFVRAQIRSGVL**TPTPTPTPTPTP**
SAAPTPTPTAGGSLVVQYRAADTNAGDNQLKPHFRIVNRGTTSVPLSELSIRYWYTVDGD
KPQVFNCDWAQVGCSNLRGSFVKLSTGRTGADYYIEITFTSGAGSLAAGGSSGDIQVRIN
KNDWTNYNEANDYSYDPTKTSFADWNRVTLYRNGQLIWGVEP

Supplementary Figure 13

Protinease K digestion of CelAC. (A) Proteinase K digestion of an AD preparation (7.5 μg, CelABC complex in box), CelA (2 μg), CelC (2 μg) and a mixture of CelA and CelC (1:1 ratio; 2 μg each). All proteins were analyzed by SDS PAGE in the presence (+) or absence (−) of Proteinase K (PK) (187.5 μg/ml at 50 °C for 1 h). MW markers in lane 1 are in kDa, and lane 2 was left blank. This gel was representative of three gels representing technical replicates of the protease reactions. The image is cropped for clarity and the original gel image is in Supplementary Figure 17.

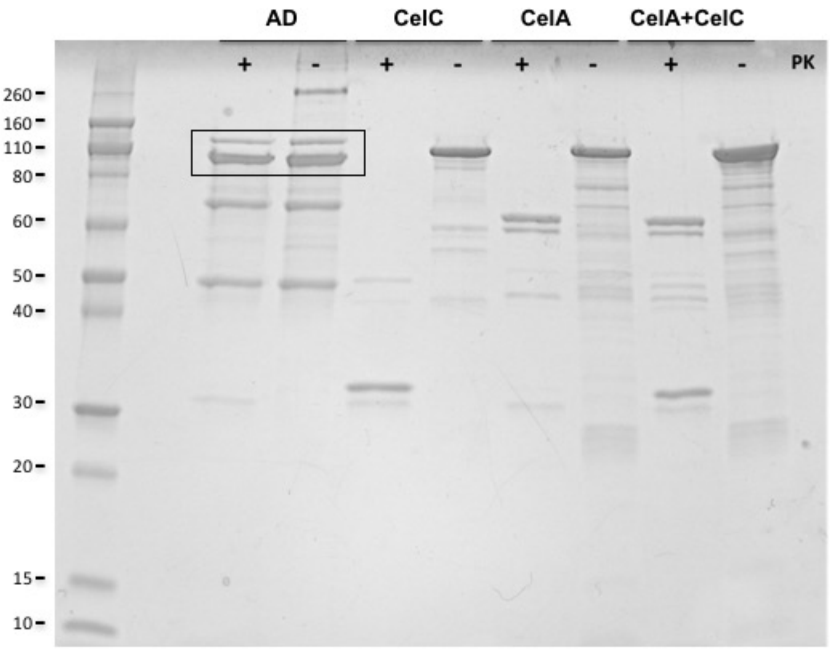

Supplementary Figure 14 Original gel images for Figure 3

Figure 3a (BN-PAGE: Coomassie/CMC zymogram/xylan zymogram)

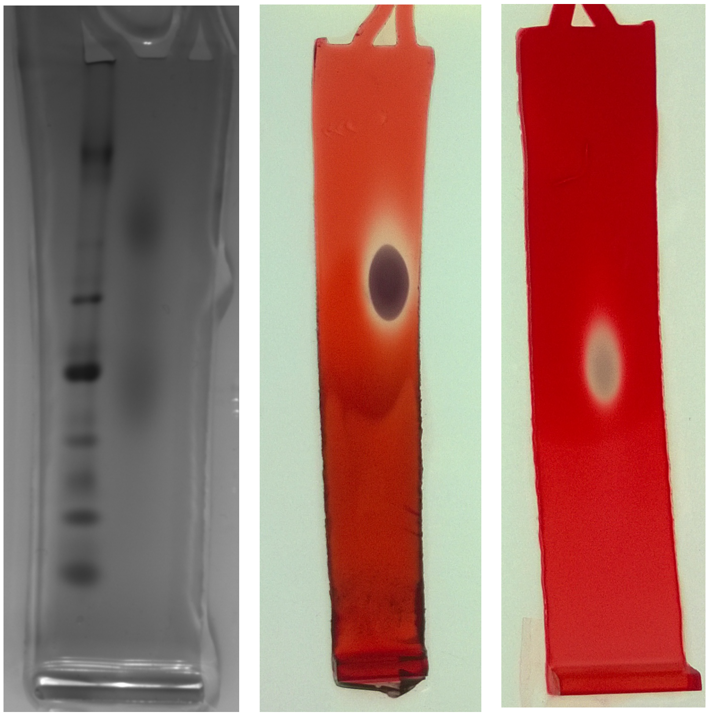

Figure 3b (2nd dimension SDS-PAGE: Coomassie/CMC zymogram)

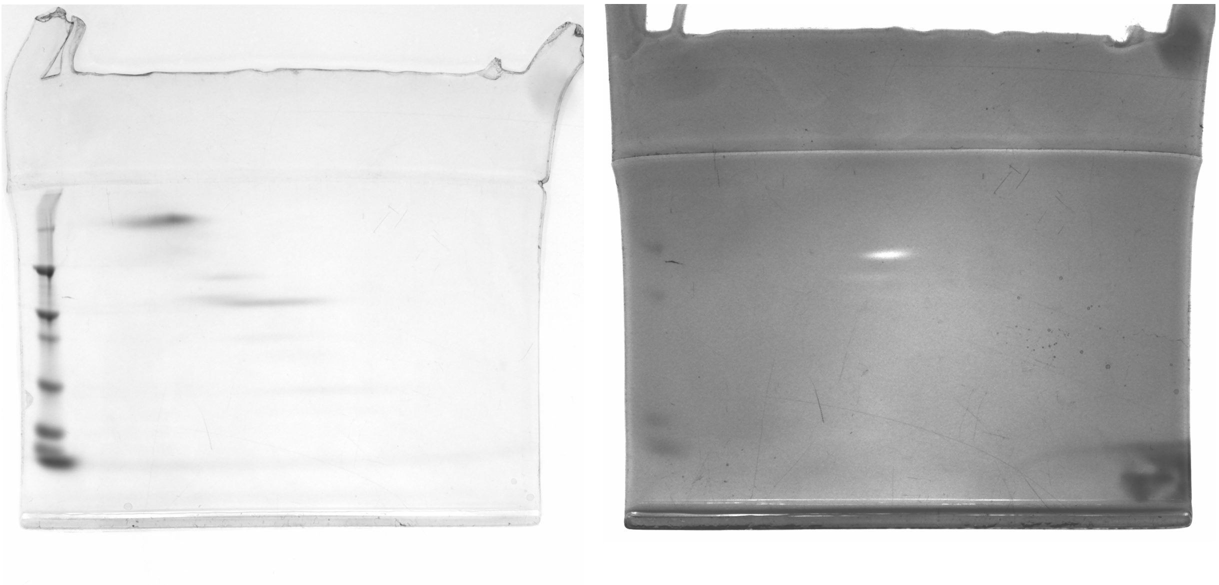

Figure 3c (BN-PAGE: Coomassie/CMC zymogram/xylan zymogram)

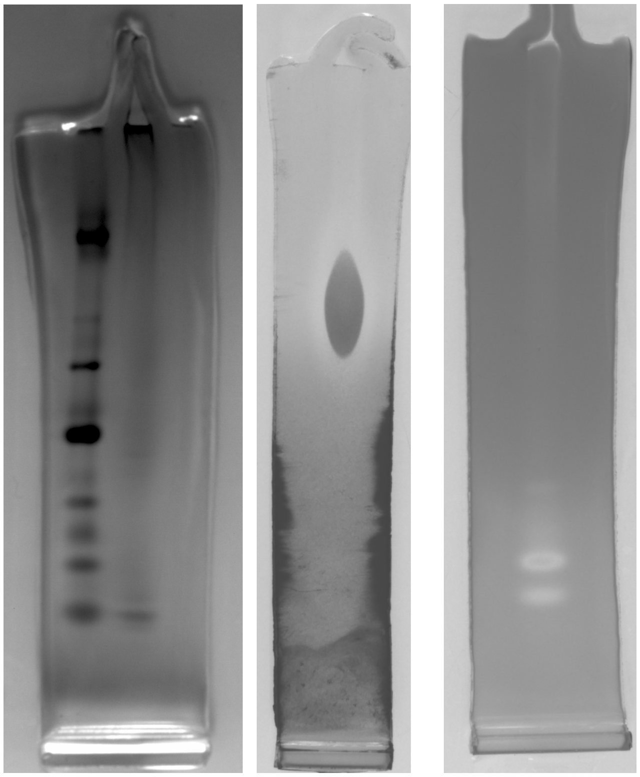

Figure 3d (2nd dimension SDS-PAGE: Coomassie/CMC zymogram)

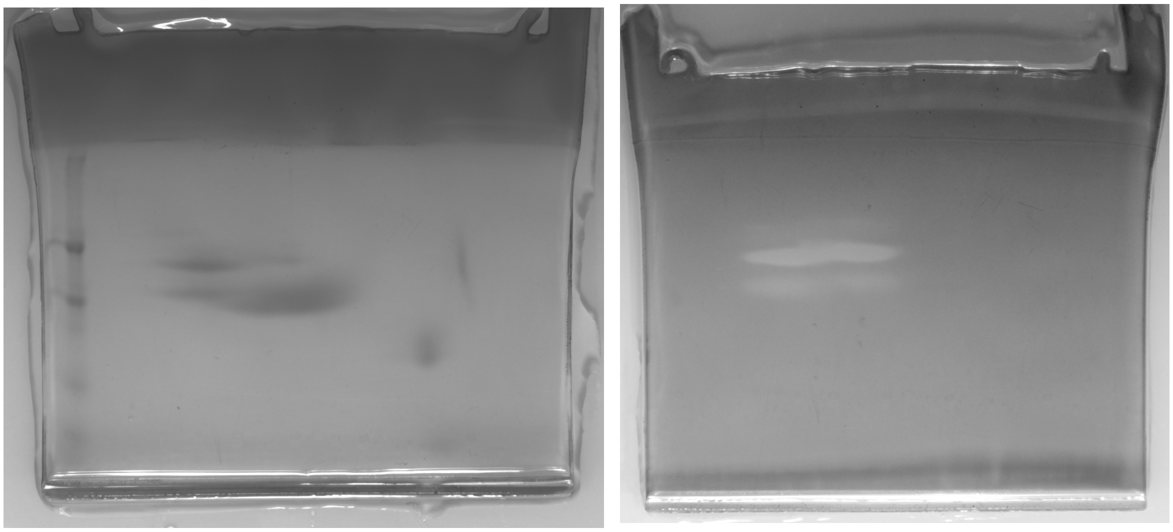

Figure 3e (2nd dimension SDS-PAGE-no heat denaturation: Coomassie/CMC zymogram)

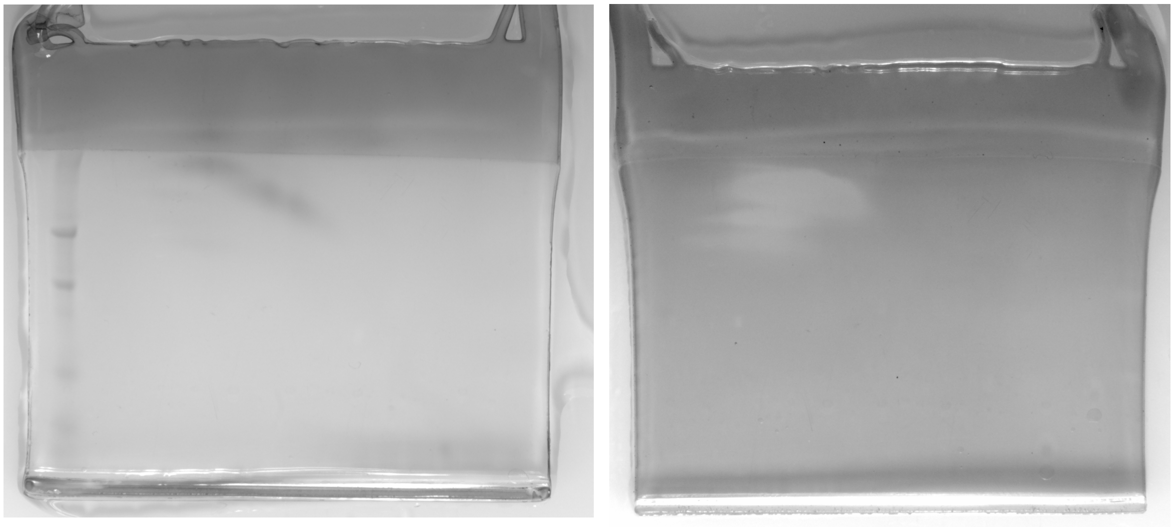

Supplementary Figure 15 Original gel images for Figure 4c. The gels were run together in one electrophoresis apparatus and overlaid to compare molecular weights accurately for the combined image.

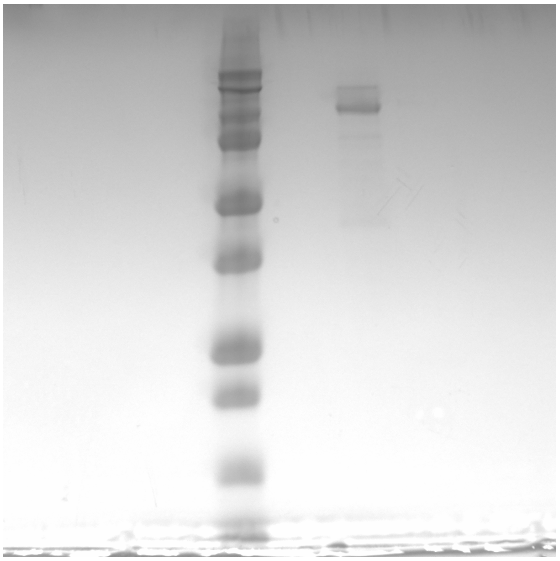

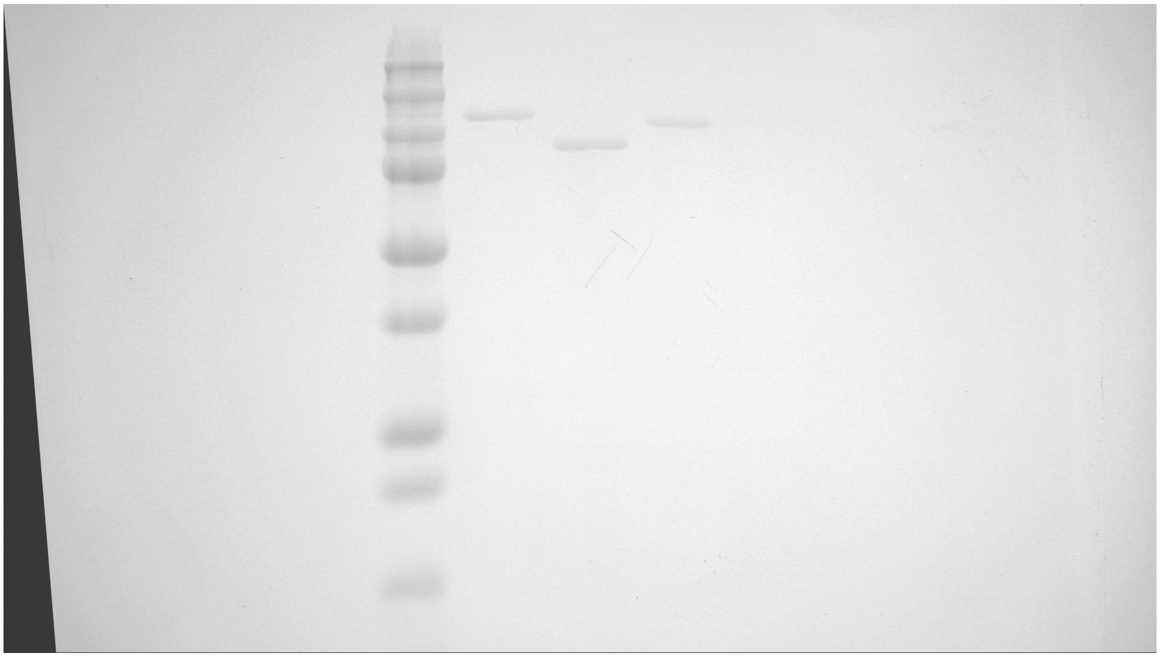

Supplementary Figure 16 Original gel image for Supplementary Figure S10. The three lanes were run in a single gel and then the last lane excised using a razor blade for periodic acid-Schiff base staining. The first two lanes were stained with Coomassie and then the three lanes aligned for the photograph.

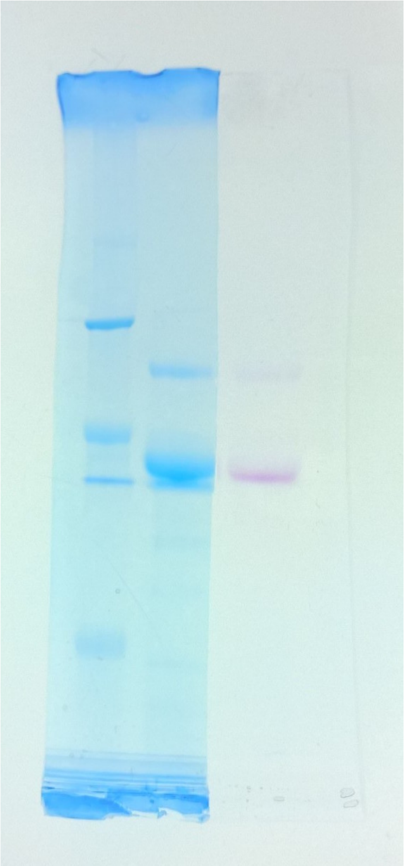

Supplementary Figure 17 Original gel image for Supplementary Figure S13.

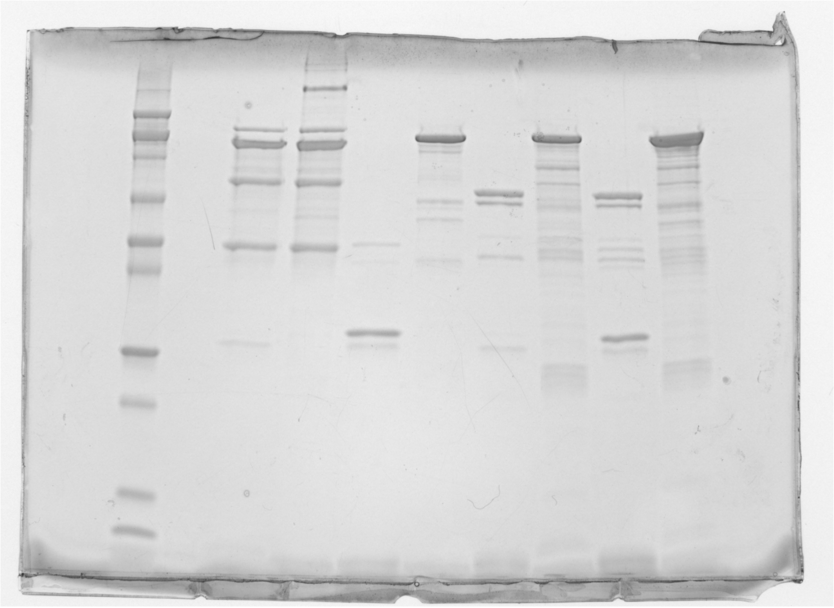

Supplement: Supplementary file 1 — Supplementary Tables 1–12 and Supplementary Figures 1–17. [file 41564_2017_52_MOESM1_ESM.pdf]
